# Supplementary material for: Global tropical dry forest extent and cover: A comparative study of bioclimatic definitions using two climatic data sets
Source: PLoS One. 2021 May 20;16(5):e0252063. doi: 10.1371/journal.pone.0252063 (PMC8136719; doi:10.1371/journal.pone.0252063)

Comparison of canopy thresholds using FAO CHELSA for tropical dry forest cover (km^2^) in 2000 and 2020 between 30˚N and 30˚S.

| **Canopy Cover** | **Forest Cover**  **2000** | **Estimated Cover**  **2020** | **Gross Loss**  **2001-2020** | **Percent Loss** |
| --- | --- | --- | --- | --- |
| Open, > 10% | 9,625,291 | 8,883,523 | 741,768 | 7.71% |
| Closed, > 40% | 4,931,414 | 4,369,695 | 561,719 | 11.39% |
| Closed, > 60% | 2,364,346 | 2,085,084 | 279,262 | 11.81% |

Comparisons of best methods for estimating tropical dry forest extent and forest cover in 2000 and 2020 with open (> 10% open canopy) and closed (> 40% closed canopy) canopies between 30˚N and 30˚S.

| **Definition** | **Source** | **Canopy** | **Forest Cover 2000** | **Estimated Cover 2020** | **Gross Loss 2001-2020** | **Percent Gross Loss** |
| --- | --- | --- | --- | --- | --- | --- |
| Pantropics | GFC | Open  Closed | 27,352,569  20,064,659 | 24,977,794  17,962,101 | 2,374,775  2,102,558 | 8.68%  10.48% |
| Biodiversity Hotspots | Hoffmann et al. 2016 | Open  Closed | 8,828,443  7,096,634 | 7,679,278  6,034,221 | 1,149,165  1,062,413 | 13.02%  14.97% |
| FAO | CHELSA | Open  Closed | 9,625,291  4,931,461 | 8,883,523  4,369,742 | 741,768  561,719 | 7.71%  11.39% |
| Murphy & Lugo | Worldclim | Open  Closed | 11,626,015  7,706,856 | 10,532,421  6,779,772 | 1,093,594  927,084 | 9.41%  12.03% |
| TSDBF | WWF | Open  Closed | 1,049,668  782,492 | 927,467  670,336 | 122,201  112,156 | 11.64%  14.33% |
| Consensus (all) | CHELSA | Open  Closed | 98,632  20,758 | 94,772  19,166 | 3,860  1,592 | 3.91%  7.67% |
|  | Worldclim | Open  Closed | 813,914  332,336 | 769,348  305,973 | 44,566  26,363 | 5.48%  7.93% |

Regional comparisons of (a) mean, (b) maximum, and (c) minimum forest cover percentage at 1 km over tropical dry forest field plots in 2000 between 30˚N and 30˚S.

(a) Mean Cover Percentage


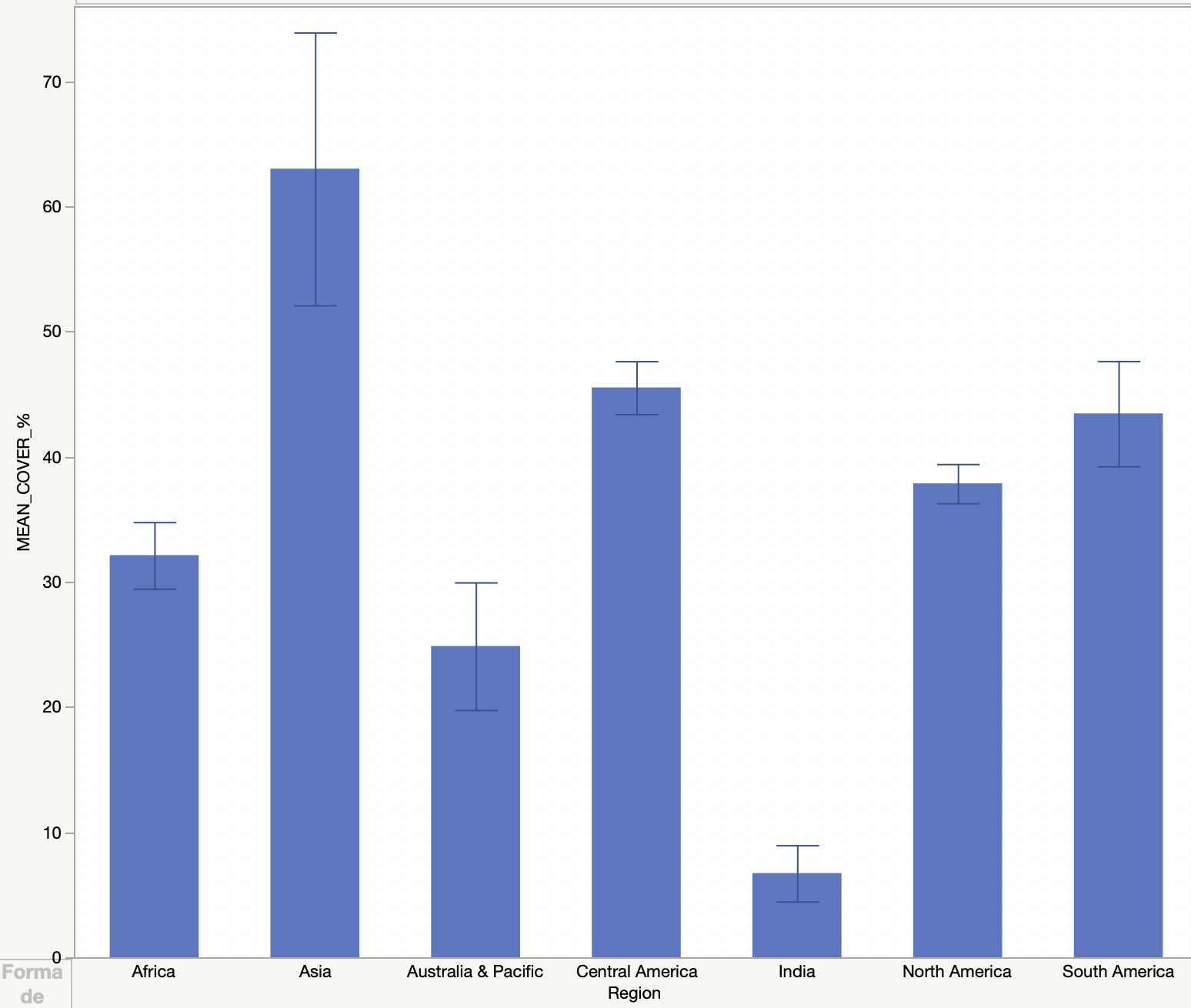


(b) Maximum Cover Percentage


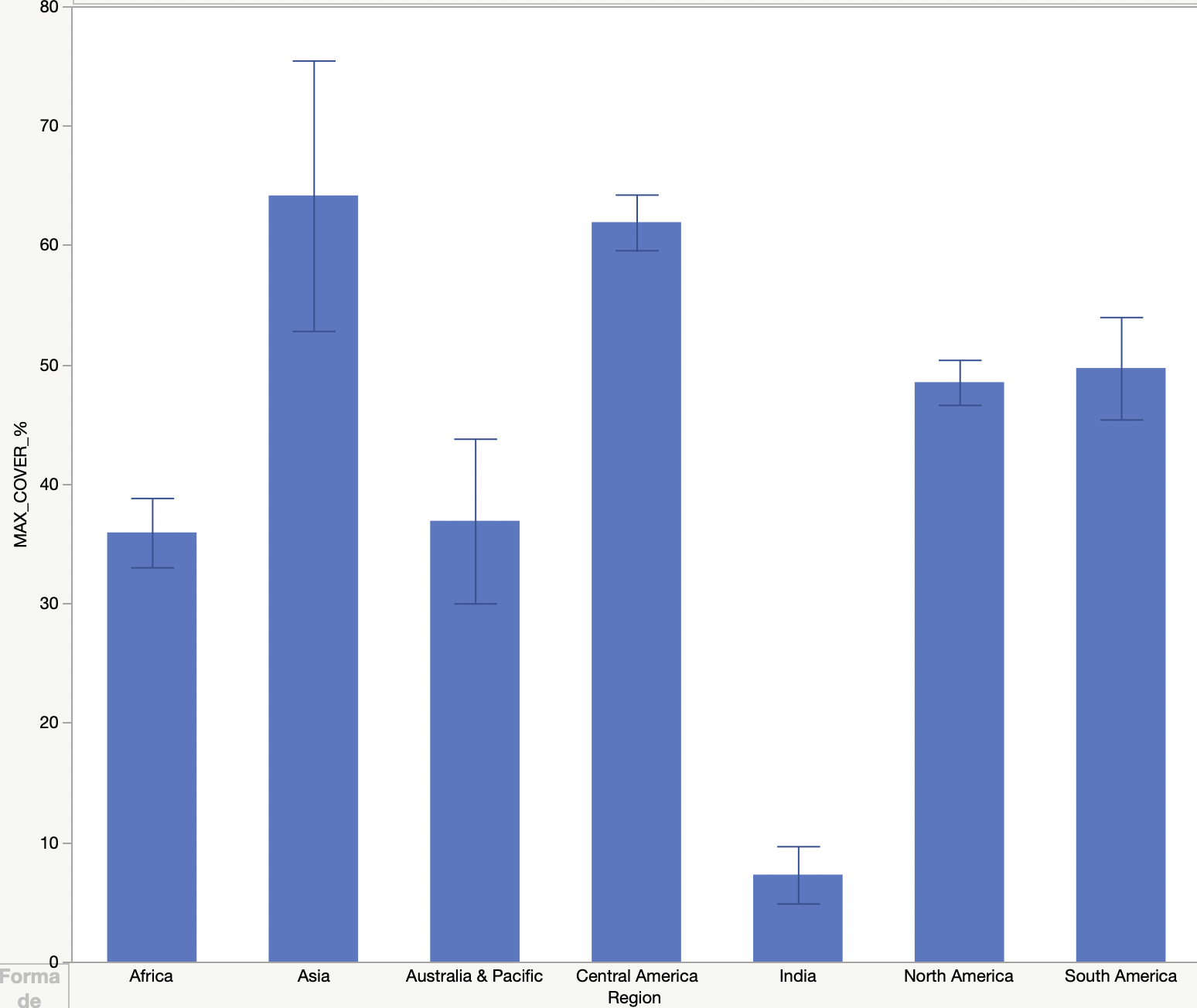


(c) Minimum Cover Percentage


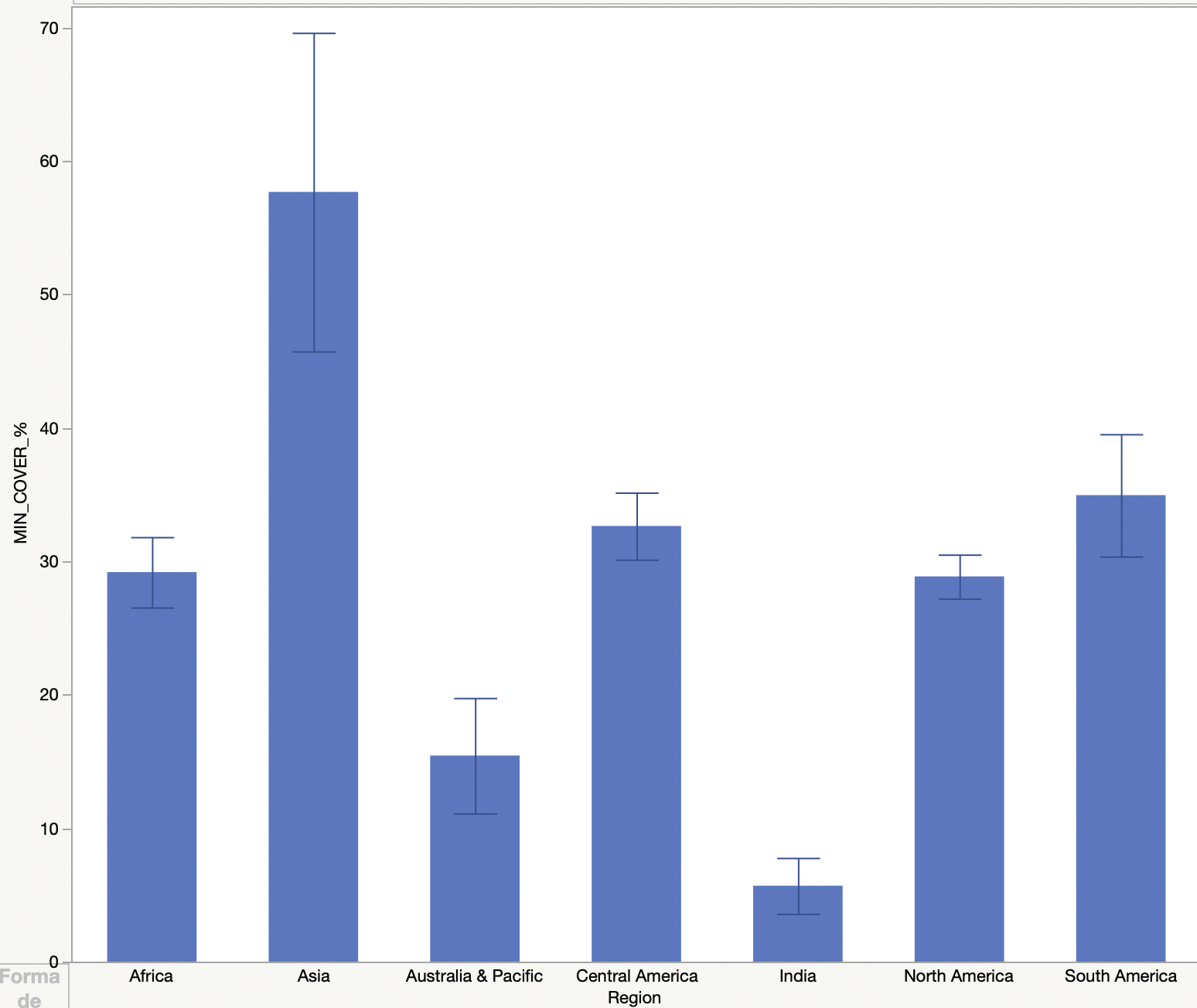

Supplement: S3 Appendix — Forest cover statistics for 540 tropical dry forest plots using resampled Global Forest Watch tree canopy cover (1 km2) data for the year 2000 and a 1 km buffer around each plot. Total forest cover estimates and gross forest cover loss are also calculated for open canopy (≥ 10%) and closed canopy (≥ 40% and ≥ 60%) for FAO CHELSA from 2000 to 2020. (DOCX) [file pone.0252063.s003.docx]
