# Supplementary material for: Global tropical dry forest extent and cover: A comparative study of bioclimatic definitions using two climatic data sets
Source: PLoS One. 2021 May 20;16(5):e0252063. doi: 10.1371/journal.pone.0252063 (PMC8136719; doi:10.1371/journal.pone.0252063)
Supplement: S6 Appendix — Code used to accomplish a global analysis of tropical dry forest extent and cover based on a suite of remote sensing and modeled climate and forest data, as well as GIS layers of political and conservation boundaries. (PDF) [file pone.0252063.s006.pdf]

S6 Appendix: Computer code for global analysis of tropical dry forest extent and cover

Jonathan P. Ocón

Overview

This appendix provides code used to accomplish a global analysis of tropical dry forest extent and cover based on a suite of remote sensing and modeled climate and forest data, as well as GIS layers of political and conservation boundaries.

A combination of geospatial modules were used across three programming languages and software to manage, analyze, and compile spatial data sets for our analysis. Free and open-source Python software (Python Software Foundation, 2019) was used to manipulate raw data to compile binary raster maps representing the fundamental layers (e.g. temperature, precipitation, seasonality) that were then overlaid on top of one another to produce a second, final binary raster for the respective climatic definition of tropical dry forest. The Python modules used most were GDAL and Rasterio.

We also lacked one raw data set (Aridity Index for CHELSA – see below), and used the ENVIronmental Rasters for Ecological Modeling (ENVIREM) R-package (Title and Bemmels, 2018) to compile a GeoTiff raster file of a global aridity index with CHELSA data. Additional R packages used in this study include the Tidyverse, which helped create all data tables, as well as Tmap, which was used in the design and compilation of all figures. All figures were compiled in a WGS84 projection.

What is not included are the house-keeping code used to set up script environments, call in modules or packages, and manage files. Only the "meat" of the data processing is included. For entire scripts, please contact the author: jonocon@g.ucla.edu.

Processing Remote Sensing and Modeled Climate Data

We collected data from three climatic data sets (Worldclim, CHELSA, Global Aridity and PET), biome defining shapefiles (WWF Ecoregions), a compilation of 579 tropical dry forest field plots, as well as the latest forest cover data to estimate the extent for tropical dry forest regions from 2000 to 2018 (Global Forest Change)(Hansen et al., 2013).

To help process large amounts of raw data, we clipped data to our study area and compressed the files by converting nodata values and datatypes.

```
#Functions for Data Compression and Clipping to Study Areas
def latlon(area):
    dset=gdal.Open(area,gdal.GA_ReadOnly)
    arr=dset.ReadAsArray()
    arr=arr.astype('int16')
    ds=gdal.Translate(area,dset,projWin=[-180.0,30.0,180.0,-30.0])
    ds=None

def compress(tif):
    with r.open(tif,'r+',compress='LZW') as dset:
        dset.nodata=0
    src=r.open(tif,'r',compress='LZW')
    meta=src.meta.copy()
    meta.update({'dtype':'uint8'
                }
               )
    arr=src.read()
    arr=arr.astype('uint8')
    with r.open(os.path.splitext(tif)[0]+
               '_compress'+'.tif',
```

```
            'w',**meta,compress='LZW') as dest:
        dest.write(arr)
```

1. Global Precipitation Measurement (GPM) IMERG L3 (Huffman et al., 2019)

We collected the Global Precipitation Measurement Integrated Multi-satellitE Retrievals for Global Precipitations Measurement (IMERG) Level 3 monthly precipitation data (mm/hour) at 0.1 deg spatial resolution (or approximately 11 km at the equator) (Huffman et al., 2019). However, the 4.25-year time series did not appear long enough to include in the analyses and contained a number of extremely high and low precipitation values.

```
#------------------------------------
#
# Extract Seasoanlity from IMERG L3 Data
#
#------------------------------------

# (1) Function to extract single band from HDF5 and save as GTiff
def hdf_band_extract(hdf,save,bnd):
    # Open HDF5
    dset=gdal.Open(hdf,gdal.GA_ReadOnly)
    band=gdal.Open(dset.GetSubDatasets()[bnd][0],gdal.GA_ReadOnly)
    # Read the band into an array
    arr=band.ReadAsArray()
    arr=arr[:,::-1]
    arr=arr.T
    # Convert nan values
    arr[arr==-9999.9]=np.nan
    # Convert to mm/day
    arr=arr*24.
    # Build save path
    fpath=os.path.join(save,os.path.basename(os.path.splitext(hdf)[0])
                   +'_B'+str(bnd+1)+'_mmday'+'.tiff')
    # Write GTiff
    tiff=gdal.GetDriverByName('GTiff').Create(fpath,
        band.RasterYSize,
        band.RasterXSize,
        1,# Number of bands
        gdal.GDT_Float32,
        ['TILED=YES'])
    srs=osr.SpatialReference()
    srs.ImportFromEPSG(4326)
    tiff.SetProjection(srs.ExportToWkt())
    tiff.SetGeoTransform(geotrans)
    tiff.GetRasterBand(1).WriteArray(arr)
    tiff.GetRasterBand(1).SetNoDataValue(-9999.9)
    tiff=None # Close file and write to 'save'

# (2) Convert from mm/day to mm/month
# Function for months with 31 days
def monthly31_avg(tif31,month):
    ds31=gdal.Open(tif31,gdal.GA_ReadOnly)
    arr=ds31.ReadAsArray()
    arr=arr*31.
    #parameter changed for 30-day months, February, and leap-years
    fpath=os.path.join(month,os.path.basename(os.path.splitext(tif31)[0])
                   +'_mmM'+'.tiff')
    tiff=gdal.GetDriverByName('GTiff').Create(fpath,
        3600, # Y-size
        1800, # X-size
        1,
        gdal.GDT_Float32,
        ['TILED=YES'])
    srs=osr.SpatialReference()
    srs.ImportFromEPSG(4326)
    tiff.SetProjection(srs.ExportToWkt())
    tiff.SetGeoTransform(geotrans)
    tiff.GetRasterBand(1).WriteArray(arr)
    tiff.GetRasterBand(1).SetNoDataValue(-9999.9)
    tiff=None

# For loop to define the files of certain months
for file in os.listdir(save):
    fname=os.fsdecode(file)
    if fname.endswith('.tiff'):
        for i in range(10):
            for j in range(10):
                if fn.fnmatch(fname,'*.%s%s.V05B_B2_mmday.tiff'%(i,j)) is True and (i,j)==(0,1):
                    tif31=os.path.join(save,fname)
                    monthly31_avg(tif31,month)
                    continue
                if fn.fnmatch(fname,'*.%s%s.V05B_B2_mmday.tiff'%(i,j)) is True and (i,j)==(0,2):
                    tif28=os.path.join(save,fname)
                    monthly28_avg(tif28,month)
                    continue
                if fname.startswith('3B-MO.MS.MRG.3IMERG.20160201-S000000-E235959.02.V05B_B2_mmday.'):
                    tif29=os.path.join(save,fname)
                    monthly29_avg(tif29,month)
                    continue
                if fn.fnmatch(fname,'*.%s%s.V05B_B2_mmday.tiff'%(i,j)) is True and (i,j)==(0,3):
                    tif31=os.path.join(save,fname)
                    monthly31_avg(tif31,month)
                    continue
                if fn.fnmatch(fname,'*.%s%s.V05B_B2_mmday.tiff'%(i,j)) is True and (i,j)==(0,4):
                    tif30=os.path.join(save,fname)
                    monthly30_avg(tif30,month)
                    continue
```

```
            if fn.fnmatch(fname,'*.%s%s.V05B_B2_mmday.tiff'%(i,j)) is True and (i,j)==(0,5):
                tif31=os.path.join(save,fname)
                monthly31_avg(tif31,month)
                continue
            if fn.fnmatch(fname,'*.%s%s.V05B_B2_mmday.tiff'%(i,j)) is True and (i,j)==(0,6):
                tif30=os.path.join(save,fname)
                monthly30_avg(tif30,month)
                continue
            if fn.fnmatch(fname,'*.%s%s.V05B_B2_mmday.tiff'%(i,j)) is True and (i,j)==(0,7):
                tif31=os.path.join(save,fname)
                monthly31_avg(tif31,month)
                continue
            if fn.fnmatch(fname,'*.%s%s.V05B_B2_mmday.tiff'%(i,j)) is True and (i,j)==(0,8):
                tif31=os.path.join(save,fname)
                monthly31_avg(tif31,month)
                continue
            if fn.fnmatch(fname,'*.%s%s.V05B_B2_mmday.tiff'%(i,j)) is True and (i,j)==(0,9):
                tif30=os.path.join(save,fname)
                monthly30_avg(tif30,month)
                continue
            if fn.fnmatch(fname,'*.%s%s.V05B_B2_mmday.tiff'%(i,j)) is True and (i,j)==(1,0):
                tif31=os.path.join(save,fname)
                monthly31_avg(tif31,month)
                continue
            if fn.fnmatch(fname,'*.%s%s.V05B_B2_mmday.tiff'%(i,j)) is True and (i,j)==(1,1):
                tif30=os.path.join(save,fname)
                monthly30_avg(tif30,month)
                continue
            if fn.fnmatch(fname,'*.%s%s.V05B_B2_mmday.tiff'%(i,j)) is True and (i,j)==(1,2):
                tif31=os.path.join(save,fname)
                monthly31_avg(tif31,month)
                continue
            else:
                continue

# Test to ensure the leap year values are unique compared to other Feb values, and that Feb values did not get processed as leap
print('Leap Year - Feb 2016, mm/month')
def test(src):
    tif=gdal.Open(src,gdal.GA_ReadOnly)
    arr=tif.ReadAsArray()
    return arr[1400,1400]
print('#1 - Leap from for loop',test(src))

# Produce a 100mm file to use for Seasonality layer
crit_100='3*.tiff'
q100=os.path.join(dir_100,crit_100)
tile100=glob.glob(q100)
t100_arr=[]

for fp in tile100:
    fname=r.open(fp,'r+')
    arr=fname.read()
    NaNs=isnan(arr)
    arr[NaNs]=0
    arr=arr/4.25
    arr_2d=arr[0,:,:]
    t100_arr.append(arr_2d)
    fname.write(arr)

t100=np.array(t100_arr)
dataOut=t100.sum(axis=0)

driver=gdal.GetDriverByName('GTiff')
dsOut=driver.Create(out_100,extent100.RasterXSize,extent100.RasterYSize,1,ex100_band.DataType)
CopyDatasetInfo(extent100,dsOut)
bandOut=dsOut.GetRasterBand(1)
BandWriteArray(bandOut,dataOut)
```

2. Worldclim (Fick and Hijmans, 2017)

The second version of the Worldclim climate data released in 2016 includes monthly temperature and precipitation measurements from 9,000 and 60,000 weather stations respectively spanning 1970 to 2000. These data were interpolated using thin-plate splines with covariates including elevation, distance to the coast and three satellite-derived covariates: maximum and minimum land surface temperature as well as cloud cover, obtained with the MODIS satellite platform (Fick and Hijmans, 2017). The data sets include the monthly temperature and precipitation averages, as well as nineteen bioclimatic variables often used in species distribution modeling. The spatial resolution of the data are 30 arc seconds or ~1 km resolution at the equator.

Layers produced with this data correspond to parameters set in each climatic definition of tropical dry forest:

- Annual precipitation (mm/year)
- Seasonality, or # of months experiencing precipitation less than 100 mm/mos
- Mean Annual Temperature (C)
- No Freeze Mask (>0C)

3. CHELSA (Karger et al., 2017)

The second climate data set we use is the Climatologies at High resolution for the Earth's Land Surface Area (CHELSA) currently hosted by the Swiss Federal Institute for Forest, Snow and Landscape Research. CHELSA includes monthly mean temperature and precipitation patterns from 1979 to 2013 and is based on a quasi-mechanistically statistical downscaling of the ERA interim global circulation model with a GPCC bias correction (Krager et al., 2017). The data spatial resolutions are also 30 arc seconds or ~1 km resolution at the equator.

The layers produced with this data match those of Worldclim (see previous section).

4. Aridity Index (Trabucco and Zomer, 2018)

Drylands and associated dry forest extent has been tested at an aridity index of less than 0.65 (Bastin et al., 2017). We compare the climatic definitions (see next section) of tropical dry forest against this aridity threshold. To test global aridity index, or the ratio of potential evapotranspiration to annual precipitation, we collected data from the Global Aridity and PET database, an addition of the Worldclim data set (Trabucco and Zomer, 2019).

```
#------------------------------------
#
# Mask monthly 100mm thresholds from WorldClim v2, CHELSA, and Aridity Index (Worldclim ONLY)
# To switch between data sets, replace the nodata value in the functions
# nodata in Worldcim = 32768, CHELSA = 32767, Aridity (Worldclim) = -2.14748e+09
#
#------------------------------------

# Define function to extract the months that are <= 100
def mon_100(mon100):
    with r.open(mon100,'r+') as dset:
        arr=dset.read()
        arr[arr==-32768]=101
        arr[arr<=100]=1 # for the Aridity Index layer, arr<=6500
        arr[arr>100]=-32768
        dset.write(arr)

# Overlap monthly 100mm into one tiff
# 100mm file
crit_100='w*.tif'
q100=os.path.join(dir100,crit_100)
tile100=glob.glob(q100)
t100_arr=[]

for fp in tile100:
    fname=r.open(fp,'r+')
    arr=fname.read()
    arr[arr==-32768]=0
    arr_2d=arr[0,:,:]
    t100_arr.append(arr_2d)
    fname.write(arr)

t100=np.array(t100_arr)
dataOut=t100.sum(axis=0)

driver=gdal.GetDriverByName('GTiff')
dsOut=driver.Create(out_100,ex100.RasterXSize,ex100.RasterYSize,1,b100.DataType)
CopyDatasetInfo(ex100,dsOut)
bandOut=dsOut.GetRasterBand(1)
BandWriteArray(bandOut,dataOut)

# Define function to extract the dry seasons for each definition
def ml(murph): # variables changed for each definition
    with r.open(murph,'r+') as dset:
        arr=dset.read()
        arr[arr==1]=0
        arr[(arr>=4)&(arr<=7)]=1 # thresholds changed for each deifnition
        arr[arr!=1]=0
        dset.write(arr)

#------------------------------------
#
# Mask AP for TDF definitions
#
#------------------------------------

# Define function to extract AP
def ML(ml):
    with r.open(ml,'r+') as dset:
        arr=dset.read()
        arr[(arr>=250)&(arr<=2000)]=1 # Specific thresholds are changed for each definition
        arr[arr!=1]=0 # creates binary raster
        dset.write(arr)

#------------------------------------
#
# Mask temperatures for TDF definitions
# Bioclimatic Variables for MAT and Lowest Temp
#
#------------------------------------

# Define function to extract temps that are >0C
```

```python
def no_frz(n0):
    with r.open(n0,'r+') as dset:
        arr=dset.read()
        arr[arr>0]=1
        arr[arr!=1]=0
        dset.write(arr)

# Define function to extract temps that are >17C
def MAT(mat):
    with r.open(mat,'r+') as dset:
        arr=dset.read()
        arr[arr==1]=0
        arr[arr>=17]=1
        arr[arr!=1]=0
        dset.write(arr)
```

ENVIREM (Title and Bemmels, 2018)

To obtain the aridity index for CHELSA data, we used the ENVIronmental Rasters for Ecological Modeling (ENVIREM) R-package (Title and Bemmels, 2018) to produce a global annual potential evapotranspiration raster file, which was then used to calculate aridity using the equation: annual precipitation / potential evapotranspiration (Bastin et al., 2017; Trabucco and Zomer, 2019).

```r
#------------------------------------
#
# Calculate PET for CHELSA data
# Computationally intensive, data is divided into 6 regions globally
#
#------------------------------------

# Split global data into North West pantropical region
# Following steps are repeated for 5 other regions
poly <- readWKT("POLYGON((
                -180.0001388889999987 29.9998606310000007,
                -30.0 29.9998606310000007,
                -30.0 0.0,
                -180.0001388889999987 0.0,
                -180.0001388889999987 29.9998606310000007
))", p4s = CRS("+proj=longlat +datum=WGS84"))

# crop and mask input rasters
inputDir <- 'C:/Users/Jonathan Ocon/Desktop/tropics'
outputDir <- 'C:/Users/Jonathan Ocon/Desktop/tropics/NW'

files <- list.files(inputDir, pattern = '.tif', full.names = TRUE)

r <- stack(files)
r <- crop(r, poly)
r <- mask(r, poly)

for (i in 1:nlayers(r)) {
  fn <- paste(outputDir, '/' , names(r)[i], '.tif', sep = '')
  writeRaster(r[[i]], filename = fn, format = 'GTiff', overwrite = TRUE)
}

## clear temp file cache
removeTmpFiles(h = 0)

## option to include different temp directory
rasterOptions(tmpdir = 'D:/tmp')

inputDir <- 'C:/Users/Jonathan Ocon/Desktop/tropics/NW'
outDir <- 'D:/envirem'

## generate envirem rasters, 'all' creates 17 additional ecological rasters
generateRasters(var = 'all' ,
                maindir = inputDir ,
                resName = '30arcsec',
                timeName = 'NW',
                outputDir = outDir ,
                rasterExt = '.tif' ,
                tempScale = 10 ,
                overwriteResults = TRUE ,
                outputFormat = 'GTiff' ,
                gdalinfoPath = NULL ,
                gdal_translatePath = NULL,
                useCompression = TRUE)

## clear temp file cache
removeTmpFiles(h = 0)

#------------------------------------
#
# Calculate Aridity Index for CHELSA data
#
#------------------------------------

## call in PET and AP rasters
pet <- raster('annualPET.tif')
ap <- raster('bio_12.tif')

## change 0 to NA in PET raster
pet[pet==0] <- NA
pet
writeRaster(pet, filename = 'annualPET.tif', format = 'GTiff', overwrite = TRUE)

## aridity index
ai <- ap/pet
writeRaster(ai, filename = 'ai.tif', format = 'GTiff')
```

Layering Climatic Definitions of Tropical Dry Forest

For all definitions, we use frost-free regions (> 0C) in the tropics that also experience a mean annual temperature > 17C (Murphy and Lugo, 1986).

1. Murphy and Lugo (Holdridge, 1967; Murphy and Lugo, 1986)

Tropical dry forest extent from Murphy and Lugo were calculated by subsetting areas with 250 to 2000 mm of annual precipitation with a dry season(s) of 4 to 7 months with less than 100 mm of precipitation a month (Holdridge, 1967; Murphy and Lugo, 1986).

2. Food and Agriculture Organization (FAO) (Sunderland et al., 2015)

Tropical dry forest extent from the Food and Agriculture Organization of the United Nations (FAO) were created by subsetting areas with 500 to 1500 mm of annual precipitation with a dry period of 5 to 8 months with less than 100 mm of precipitation a month (Sunderland et al., 2015).

3. DryFlor (Banda et al., 2016)

Tropical dry forest extent from DryFlor were calculated as annual precipitation less than 1800 mm with a dry season of 3 to 6 months receiving less than 100 mm per month (Banda et al., 2016).

The following code is for overlaying the necessary climatic layers for Murphy and Lugo definition of tropical dry forest. It can be adapted for FAO and DryFlor./

```
# Define function to extract the dry seasons for each definition
def ml(murph): # variables differ for each respective definition
    with r.open(murph,'r+') as dset:
        arr=dset.read()
        arr[arr==1]=0
        arr[(arr>=4)&(arr<=7)]=1 # dry season depends on definition
        arr[arr!=1]=0
        dset.write(arr)

# Murphy and Lugo -- changed for FAO, DryFlor, AI
crit_ml='*ML.tif'
q_ml=os.path.join(climate,crit_ml)
tile_ml=glob.glob(q_ml)
t_ml_arr=[]

for fp in tile_ml:
    fname=r.open(fp,'r+')
    arr=fname.read()
    arr[arr==-1.7e+308]=0
    arr_2d=arr[0,:,:]
    t_ml_arr.append(arr_2d)
    fname.write(arr)

t_ml=np.array(t_ml_arr)
dataOut=t_ml.sum(axis=0)

driver=gdal.GetDriverByName('GTiff')
dsOut=driver.Create(out_murph,ex.RasterXSize,ex.RasterYSize,1,b.DataType)
CopyDatasetInfo(ex,dsOut)
bandOut=dsOut.GetRasterBand(1)
BandWriteArray(bandOut,dataOut)

ex=None
bandOut=None
dsOut=None

#Run function to mask 3 settings to 1
def final_murph(out_murph):
    with r.open(out_murph,'r+') as dset:
        arr=dset.read()
        arr[arr!=3]=0
        arr[arr==3]=1
        dset.write(arr)

final_murph(out_murph)
```

Regional Tropical Dry Forest Extent and Cover

Originally launched to provide high-resolution global maps of forest cover change from 2000 – 2012 using Landsat 7 imagery, the Global Forest Change data set has grown to include time series analysis of Landsat 5, 7 and Landsat 8 imagery now covering 2000 – 2018 (Hansen et al., 2013). Each pixel has a spatial resolution of 1 arc second, or roughly 30 m, and unsigned 8-bit values (0-255). We identify areas with > 10% cover

(encompasses mosaic of savannas and woodlands) and > 40% cover or forest for the year 2000 and 2018 (Bastin et al., 2017). We utilize this as our primary data set to calculate tropical dry forest cover and loss.

We calculate pantropical results within 30N and 30S globally. We divided global results into six macro-scale regions (Africa, North and Central America, South America, South Asia, and South East Asia and Asia Pacific). We further sub-divided regions using Biodiversity Hotspots (Myers et al., 2000; Hoffman et al., 2016) and countries (Natural Earth, 2019) for a meso-scale analyses (Appendix 3 and 4). Tropical dry forests on islands are generally combined in global analyses (Miles et al., 2006; Schmitt et al., 2009), however, these islands contain the smallest extents and fragments of tropical dry forest, which emphasizes the need to report the extent of the tropical dry forest on islands to assess global conservation priorities (Sunderland et al., 2015). Thus, we select tropical four island archipelagos (Fiji, Galapagos, Hawai'i, Puerto Rico) as an example of micro-scale archipelago analyses. Tropical dry forest biome and forest types are well known in Puerto Rico and Hawai'i, while Fiji is on the wetter end of the spectrum and the Galapagos is on the drier end.

WWF Ecoregions (Olson et al., 2001)

The ecoregions polygon shapefile includes 867 land units classified into 14 different biomes such as forests, grasslands, or deserts (Olson et al., 2001; WWF, 2019). Each polygon also represents areas of land with an assemblage of species, dynamics, and environmental conditions (WWF, 2019). WWF has identified 53 ecoregions that fall within the Tropical and Subtropical Dry Broadleaf Forest category (Appendix 1). We test the validity of these 53 WWF Ecoregions, but acknowledge that previous studies have established the presence of tropical dry forest in other WWF Ecoregion biomes such as Tropical and Subtropical Grasslands, Savannas and Shrublands (Miles et al. 2006). We calculated the area of each WWF Ecoregion (Appendix 1) and compare results with four climatic definitions. We identified the proportion of overlap between WWF Ecoregions and four climatic definitions, which identifies areas where there are overlap with WWF Ecoregions, as well as potential tropical dry forest extent outside of the 53 WWF ecoregions.

Subcontinents, Countries, Island Archipelagos (Natural Earth, 2019) and Biodiversity Hotspots (Hoffman et al., 2016)

To analyze this regional data, we organized the (1) area calculations (python), (2) overlaps (R), and percentages into (3) excel tables (see List of Tables section in Manuscript).

(1) Area Calculations:

```
#--------------------
#
#(1)Create 7200x43200 array filled with desired areas for deg to km2
#   Every row, values increase from 742249591 to 85734000 in 3191 increments then back to 742249591
#(2)Sum both arrays and all values that == 742249591 ... 85734000 = 0, convert to int32
#(3)Clip global rasters to each shapefile
#(4)Total all remaining values in each file
#
#--------------------

#(1) Create an array matching 30N/S clip full of accurate area values for each row
print('Creating 30N/S array for deg to km2 area conversion...')
#Fill an array with desired area values
a=np.arange(74249591,85737191,3191)
b=np.arange(85734000,74246400,-3191)
c=np.append(a,b)
d=np.array([c,c])
e=np.transpose(d)

#Duplicate the second column 43198 times for 7200x43200 array
def dup_cols(e,indx,num_dups=1):
    return np.insert(e,[indx+1]*num_dups,e[:,[indx]],axis=1)

f=dup_cols(e,indx=1,num_dups=43198)

#(2) Call in each raster and sum with the custom array above, any values == original custom vals = 0
def km(area):
    with rasterio.open(area,'r+',compress='LZW') as dset:
        arr=dset.read()
        arr=arr.astype(rasterio.float64)
        arr=arr+f
        arr[arr==f]=0
        with rasterio.Env():
            profile=dset.profile
            profile.update(
                dtype=rasterio.float64)
            with rasterio.open(area,'w',**profile) as dset:
                dset.write(arr)

#(3) Call in each raster, clip to each shapefile
#Raster criteria
```

```
crit_rast='*.tif'
q_rast=os.path.join(rast,crit_rast)
tile_rast=glob.glob(q_rast)

#MACRO -- repeated for each region!
crit_macro='*.shp'
q_macro=os.path.join(macro,crit_macro)
tile_macro=glob.glob(q_macro)

def clip_macro(raster):
    for fp in tile_macro:
        if fp.endswith('.shp'):
            shp=fp
            print('Clipping '+raster+' to '+shp+' ...')
            with fiona.open(shp,'r') as shapefile:
                features=[feature['geometry'] for feature in shapefile]
            with rasterio.open(raster) as src:
                out_image,out_transform=rasterio.mask.mask(src,features,
                                                    crop=True)
                out_meta=src.meta.copy()
                out_meta.update({'driver':'GTiff',
                                 'height':out_image.shape[1],
                                 'width':out_image.shape[2],
                                 'transform':out_transform})
                with rasterio.open(os.path.join(FINAL,os.path.basename(os.path.splitext(raster)[0])+'_'+os.path.basename(os.path.splitext(shp)[0])+'_FINAL_macro'+'.tif'),'w',**out_meta) as dest:
                    dest.write(out_image)
            print(raster+' clipped.')
            print()
            continue
        else:
            continue

#(4) Run raster stats on each file to determine the area and output each result to excel file
crit_final='*.tif'
q_final=os.path.join(FINAL,crit_final)
tile_final=glob.glob(q_final)
tfinal_arr=[]

def stats(fp):
    with rasterio.open(fp,'r+',compress='LZW') as src:
        arr=src.read(1,masked=True) # covers up nan vals
        arr=arr/1e8
        if np.sum(arr)>=0:
            tfinal_arr.append([fp,np.sum(arr)])
            src=None
```

(2) Overlaps:

```
# create a list of tifs -- CHELSA -- stack, sum, plot, write file to cwd
list_ch <- list.files(cwd , pattern = "*ch-clip.tif$")
stack_ch <- raster::stack(list_ch)
sum_ch <- sum(stack_ch , na.rm = TRUE)
plot(sum_ch)
writeRaster(sum_ch , 'tdf_overlap_ch' , format = 'GTiff')

# repeat for Worldclim
list_wc <- list.files(cwd , pattern = "*wc-clip.tif$")
stack_wc <- raster::stack(list_wc)
sum_wc <- sum(stack_wc , na.rm = TRUE)
plot(sum_wc)
writeRaster(sum_wc , 'tdf_overlap_wc', format = 'GTiff')

# repeat for Aridity Index
list_ai <- list.files(cwd , pattern = "AI")
stack_ai <- raster::stack(list_ai)
sum_ai <- sum(stack_ai , na.rm = TRUE)
plot(sum_ai)
writeRaster(sum_ai , 'tdf_overlap_ai', format = 'GTiff')
```

(3) Excel Tables:

```
## read in plot and area csv's produced from python and QGIS
cntry <- read_csv('tdf_cntry_areas.csv')
htspt <- read_csv('tdf_htspt_areas.csv')
cntry_plots <- read_csv('countries-plots.csv')
htspt_plots <- read_csv('hotspots-plots.csv')

## remove unwanted words from filenaming
stpwrds=c('tif',
          '.',
          'cntry',
          'htspt',
          'FINAL',
          'SOVEREIGNT',
          'NAME',
          'area',
          'clip')

## break apart filenaming - 'climate dataset' & 'definition' + 'region'
x <- cntry$Region %>%
  str_replace_all("-","- ") %>%
  str_replace_all("_","- ") %>%
  removeWords(stpwrds) %>%
  str_remove_all("-")

y <- htspt$Region %>%
  str_replace_all("-","- ") %>%
  str_replace_all("_","- ") %>%
  removeWords(stpwrds) %>%
  str_remove_all("-")

## add as a new col
```

```r
cntry$names <- x
htspt$names <- y

## remove previous col
cntry <- cntry %>%
  select(-Region) %>%
  group_by(names)

htspt <- htspt %>%
  select(-Region) %>%
  group_by(names)

#### (TABLE 2) CLIMATE DEFS ####
## all climate extents excluding ecoregions
table_2 <- areas %>%
  filter_all(any_vars(str_detect(. , 'wc') | str_detect(. , 'ch'))) %>%
  filter_all(all_vars(!grepl('wwf' , .) & !grepl('tropics' , .)))

# Regions
defs_table_2 <- c("wc" , "ch" , "65" , "ml" , "fao" , "dry")
d_no <- table_2$names %>%
  removeWords(defs_table_2)
table_2$Region <- d_no

# ML
table_2$Murphy_and_Lugo <- ifelse(grepl("ml ch" , table_2$names) , table_2$Area ,
                                  ifelse(grepl("ml wc" , table_2$names) , table_2$Area , NA))
# FAO
table_2$FAO <- ifelse(grepl("fao ch" , table_2$names) , table_2$Area ,
                      ifelse(grepl("fao wc" , table_2$names) , table_2$Area , NA))
# Dryflor
table_2$Dryflor <- ifelse(grepl("dry ch" , table_2$names) , table_2$Area ,
                          ifelse(grepl("dry wc" , table_2$names) , table_2$Area , NA))
# AI
table_2$Aridity <- ifelse(grepl("ch 65" , table_2$names) , table_2$Area ,
                          ifelse(grepl("wc 65" , table_2$names) , table_2$Area , NA))

## relevel the order of Region col
table_2$Region <- str_squish(table_2$Region)
Regions <- c("Global" ,
             "Africa" ,
             "North and Central America" ,
             "South America" ,
             "South Asia" ,
             "South East Asia and Asia Pacific" ,
             "Caribbean" ,
             "East Melanesian" ,
             "Madagascar and Indian Ocean" ,
             "New Caledonia" ,
             "Polynesia and Micornesia" ,
             "Sundaland and Nicobar of India" ,
             "Tumbes Choco Magdalena" ,
             "Wallacea" ,
             "Fiji" ,
             "Hawaii" ,
             "Galapagos" ,
             "Puerto Rico")

## create final dataframe and print to csv
table_2 <- table_2 %>%
  select(-Area) %>%
  ungroup() %>%
  select(-names) %>%
  mutate(Region = fct_relevel(Region, Regions)) %>%
  arrange(Region) %>%
  group_by(Region) %>%
  summarize_all(~paste(unique(na.omit(.)) , collapse = ',')) %>%
  separate_rows(Murphy_and_Lugo , FAO , Dryflor , Aridity , convert = TRUE) %>%
  write_csv('table_2_extent-km2.csv')

######################################################
#### ECOREGIONS ####
## all climate extents in ecoregions
ecos <- areas %>%
  filter_all(any_vars(str_detect(. , 'ch') & str_detect(. , 'wwf') | str_detect(. , 'wc') & str_detect(. , 'wwf'))) %>%
  filter_all(all_vars(!grepl('tropics' , .)))
# Regions
defs_ecos <- c("ch" , "wc" , "65" , "ml" , "fao" , "dry" , "wwf")
d <- ecos$names %>%
  removeWords(defs_ecos)
ecos$Region <- d
# ML
ecos$Murphy_and_Lugo <- ifelse(grepl("ml ch" , ecos$names) , ecos$Area ,
                                  ifelse(grepl("ml wc" , ecos$names) , ecos$Area , NA))
# FAO
ecos$FAO <- ifelse(grepl("fao ch" , ecos$names) , ecos$Area ,
                      ifelse(grepl("fao wc" , ecos$names) , ecos$Area , NA))
# Dryflor
ecos$Dryflor <- ifelse(grepl("dry ch" , ecos$names) , ecos$Area ,
                          ifelse(grepl("dry wc" , ecos$names) , ecos$Area , NA))
# AI
ecos$Aridity <- ifelse(grepl("ch 65" , ecos$names) , ecos$Area ,
                          ifelse(grepl("wc 65" , ecos$names) , ecos$Area , NA))

## relevel the order of Region col
ecos$Region <- str_squish(ecos$Region)
Regions_ecos <- c("Global" ,
                  "Africa" ,
                  "North and Central America" ,
                  "South America" ,
                  "South Asia" ,
                  "South East Asia and Asia Pacific" ,
                  "Caribbean" ,
                  "East Melanesia" ,
                  "Madagascar and Indian Ocean" ,
                  "New Caledonia" ,
                  "Polynesia and Micronesia" ,
```

```r
                  "Sundaland and Nicobar of India" ,
                  "Tumbes Choco Magdalena" ,
                  "Wallacea" ,
                  "Fiji" ,
                  "Galapagos" ,
                  "Hawaii" ,
                  "Puerto Rico")

## create final dataframe and print to csv
ecos <- ecos %>%
  select(-Area) %>%
  ungroup() %>%
  select(-names) %>%
  mutate(Region = fct_relevel(Region, Regions_ecos)) %>%
  arrange(Region) %>%
  group_by(Region) %>%
  summarize_all(~paste(na.omit(.), collapse = ',')) %>%
  separate_rows(Murphy_and_Lugo , FAO , Dryflor , Aridity , convert = TRUE) %>%
  write_csv('ecoregion_extent-km2.csv')

#####################################################
#### GLOBAL LAND AREAS ####
## no climate extents, just land areas
land <- areas %>%
  filter_all(all_vars(!grepl('ch' , .) & !grepl('wc' , .)))

## relevel the order of names col - needed to peek 'l' for spaces
land$Region <- land$names %>%
  removeWords("land") %>%
  removeWords("tropics") %>%
  str_squish()

Regions_land <- c("Global" ,
                  "Africa" ,
                  "North and Central America" ,
                  "South America" ,
                  "South Asia" ,
                  "South East Asia and Asia Pacific" ,
                  "Caribbean" ,
                  "East Melanesian" ,
                  "Madagascar and Indian Ocean" ,
                  "New Caledonia" ,
                  "Polynesia and Micornesia" ,
                  "Sundaland and Nicobar of India" ,
                  "Tumbes Choco Magdalena" ,
                  "Wallacea" ,
                  "Fiji" ,
                  "Galapagos" ,
                  "Hawaii" ,
                  "Puerto Rico" ,
                  "wwf Global" ,
                  "wwf Africa" ,
                  "wwf North and Central America" ,
                  "wwf South America" ,
                  "wwf South Asia" ,
                  "wwf South East Asia and Asia Pacific" ,
                  "wwf Caribbean" ,
                  "wwf East Melanesia" ,
                  "wwf Madagascar and Indian Ocean" ,
                  "wwf New Caledonia" ,
                  "wwf Polynesia and Micronesia" ,
                  "wwf Sundaland and Nicobar of India" ,
                  "wwf Tumbes Choco Magdalena" ,
                  "wwf Wallacea" ,
                  "wwf Fiji" ,
                  "wwf Galapagos" ,
                  "wwf Hawaii" ,
                  "wwf Puerto Rico")

## create final dataframe and print to csv
land <- land %>%
  ungroup() %>%
  mutate(Region = fct_relevel(Region, Regions_land)) %>%
  arrange(Region) %>%
  select(-names) %>%
  write_csv('total_land_areas.csv')

#####################################################
#### (TABLE 3) ECOREGION AGREEMENT ####
## calculate the overlap of total ecoregion area and definition
table_3 <- ecos
table_3$total_ecos_area <- rep(land$Area[19:36], each = 2)

table_3 <- table_3 %>%
  mutate(ML_ecos_agree = (Murphy_and_Lugo / total_ecos_area * 100) ,
         FAO_ecos_agree = (FAO / total_ecos_area * 100) ,
         Dry_ecos_agree = (Dryflor / total_ecos_area * 100) ,
         AI_ecos_agree = (Aridity / total_ecos_area * 100)) %>%
  mutate_if(is.numeric, round, digits = 0) %>%
  select(-Murphy_and_Lugo) %>%
  select(-FAO) %>%
  select(-Dryflor) %>%
  select(-Aridity)

table_3[is.na(table_3)] <- 0
write_csv(table_3 , 'table_3_ecos_agree.csv')

#####################################################
#### (TABLE 4) TDF PLOT AGREEMENT ####
## in order to match rows/cols, had to manually add 'Galapagos' + 'Sundaland'
## and 'Global' (which included replacing missing areas -
## i.e. Fiji, Galapagos, and East Melanesian) to 'areas' csv
clim_plots <- clim_counts %>%
  group_by(Region) %>%
  select(-c(LAT , LON ,
         wc65 , ch65 , mlwc , mlch , faowc , faoch , drywc , drych ,
         COUNTRY , MACRO , MESO , MICRO , REF , DATABASE)) %>%
```

```
  gather(defs , value , ALL:DRY_CH) %>%
  unite("Regions" , Region:defs , remove = TRUE) %>%
  filter_all(all_vars(!grepl('NA_' , .))) %>%
  ungroup()

## break apart filenaming - 'climate dataset' & 'definition' + 'region'
defs_clim_plots <- c("WC" , "CH" , "65" , "ML" , "FAO" , "DRY" , "ALL")
clim_plots$Region <- clim_plots$Regions %>%
  str_replace_all("_","- ") %>%
  removeWords(defs_clim_plots) %>%
  str_remove_all("-")

# Global
clim_plots$All <- ifelse(grepl("ALL" , clim_plots$Regions) , clim_plots$value ,
                            ifelse(grepl("ALL" , clim_plots$Regions) , clim_plots$value , NA))
#ML
clim_plots$Murphy_and_Lugo <- ifelse(grepl("_ML_CH" , clim_plots$Regions) , clim_plots$value ,
                                  ifelse(grepl("_ML_WC" , clim_plots$Regions) , clim_plots$value , NA))
# FAO
clim_plots$FAO <- ifelse(grepl("_FAO_CH" , clim_plots$Regions) , clim_plots$value ,
                                     ifelse(grepl("_FAO_WC" , clim_plots$Regions) , clim_plots$value , NA))
# Dryflor
clim_plots$Dryflor <- ifelse(grepl("_DRY_CH" , clim_plots$Regions) , clim_plots$value ,
                                     ifelse(grepl("_DRY_WC" , clim_plots$Regions) , clim_plots$value , NA))
# AI
clim_plots$Aridity <- ifelse(grepl("_CH_65" , clim_plots$Regions) , clim_plots$value ,
                                     ifelse(grepl("_WC_65" , clim_plots$Regions) , clim_plots$value , NA))

## relevel the order of Region col
clim_plots$Region <- str_squish(clim_plots$Region)
Regions <- c("Global" ,
             "Africa" ,
             "North and Central America" ,
             "South America" ,
             "South Asia" ,
             "South East Asia and Asia Pacific" ,
             "Caribbean" ,
             "East Melanesia" ,
             "Madagascar and Indian Ocean" ,
             "New Caledonia" ,
             "Polynesia and Micronesia" ,
             "Sundaland and Nicobar" ,
             "Tumbes Choco Magdalena" ,
             "Wallacea" ,
             "Fiji" ,
             "Galapagos" ,
             "Hawaii" ,
             "Puerto Rico")

clim_plots <- clim_plots %>%
  ungroup() %>%
  select(-Regions) %>%
  select(-value) %>%
  mutate(Region = fct_relevel(Region, Regions)) %>%
  arrange(Region) %>%
  group_by(Region) %>%
  summarize_all(~paste(na.omit(.) , collapse = ',')) %>%
  separate_rows(All , Murphy_and_Lugo , FAO , Dryflor , Aridity , convert = TRUE) %>%
  write_csv('clim_plots.csv')

## create final dataframe and print to csv
table_4 <- table_3

table_4$Clim_plots <- rep(clim_plots$All[1:36])
table_4$Ecos_plots <- rep(ecos_plots$count[1:18] , each = 2)
table_4$ML_plots <- rep(clim_plots$Murphy_and_Lugo[1:36])
table_4$FAO_plots <- rep(clim_plots$FAO[1:36])
table_4$Dry_plots <- rep(clim_plots$Dryflor[1:36])
table_4$AI_plots <- rep(clim_plots$Aridity[1:36])

table_4 <- table_4 %>%
  mutate(Eco_plot_agree = (Ecos_plots / Clim_plots * 100) ,
         ML_plot_agree = (ML_plots / Clim_plots * 100) ,
         FAO_plot_agree = (FAO_plots / Clim_plots * 100) ,
         Dry_plot_agree = (Dry_plots / Clim_plots * 100) ,
         AI_plot_agree = (AI_plots / Clim_plots * 100)) %>%
  mutate_if(is.numeric, round, digits = 0) %>%
  select(-total_ecos_area) %>%
  select(-ML_ecos_agree) %>%
  select(-FAO_ecos_agree) %>%
  select(-Dry_ecos_agree) %>%
  select(-AI_ecos_agree) %>%
  select(-Ecos_plots) %>%
  select(-ML_plots) %>%
  select(-FAO_plots) %>%
  select(-Dry_plots) %>%
  select(-AI_plots)

table_4[is.na(table_4)] <- 0
write_csv(table_4 , 'table_4_plots_agree.csv')

####################################################
#### (TABLES 5-7) BIODIVERSITY HOTSPOTS ####
## HOTSPOT COUNTS
bio_2 <- table_2 %>%
  slice(13:28) %>%
  write_csv('bio_2_extent-km2.csv')

## HOTSPOT ECOS AGREEMENT
bio_3 <- table_3 %>%
  slice(13:28) %>%
  write_csv('bio_3_ecos_agree.csv')

## HOTSPOT PLOT AGREEMENT
bio_4 <- table_4 %>%
  slice(13:28) %>%
  write_csv('bio_4_plots_agree.csv')
```

Global Forest Cover (Hansen et al., 2013)

To analyze and calculate global tropical dry forest cover (Hansen et al., 2013), we used the cloud-based geospatial analysis platform Google Earth Engine (Gorelick et al. 2017). The advantage to using Earth Engine is the computing power needed to process large amounts of forest cover data (over 500 GB at pantropical scale).

```
////////////////////////////////////////////////////////////////////////////////////
//Areas of interest (.shp)//
///////////////////////////////////////////////////////////////////////////////////

//var wwf = ee.FeatureCollection("users/jonocon/tdf/wwf_terr_ecos-TDF");
//var bio = ee.FeatureCollection("users/jonocon/tdf/bio_hotspots");

var tropics = ee.Geometry.Polygon(
        [[[-180.0, 30.0],
          [-180.0, -30.0],
          [180.0, -30.0],
          [180.0, 30.0]]],null,false);


////////////////////////////////////////////////////////////////////////////////////
//Hansen Global Forest Cover//
///////////////////////////////////////////////////////////////////////////////////


//Hansen GFC
var gfc = ee.Image("UMD/hansen/global_forest_change_2020_v1_8");
var vis_10 = {'min': 0, 'max': 1, 'palette': '000000,32CD32'};
var vis_40 = {'min': 0, 'max': 1, 'palette': '000000,32CD32'};
var vis_10_loss = {'min': 0, 'max': 1, 'palette': '000000,FF530D'};
var vis_40_loss = {'min': 0, 'max': 1, 'palette': '000000,FF530D'};

// Select the treecover, lossyear bands
var land = gfc.select(['datamask']);
var treecover = gfc.select(['treecover2000']);
var lossyear = gfc.select(['lossyear']);
var loss2020 = lossyear.gte(1).and(lossyear.lte(20));

//Add the land, water
var vis_land = {'min': 0, 'max': 2, 'palette': '2F4F4F,000000,2F4F4F'};
//Map.addLayer(land, vis_land);

//Mask 10% canopy
var gfc10 = treecover.gte(10).and(treecover.lte(100));
//mask the forest covers
var gfc10_00 = gfc10.updateMask(treecover);
var gfc10_20 = gfc10.updateMask(loss2020);

//Mask 40% canopy
var gfc40 = treecover.gte(40).and(treecover.lte(100));
//mask the forest covers
var gfc40_00 = gfc40.updateMask(treecover);
var gfc40_20 = gfc40.updateMask(loss2020);


////////////////////////////////////////////////////////////////////////////////////
//Clim Defs (.tif)//
///////////////////////////////////////////////////////////////////////////////////

// ended up using exact same code, just switched the files in var FAO
// Definitions
var FAO = ee.Image("users/jonocon/tdf/FAO_CH");

var fao40_00 = FAO.updateMask(gfc40_00);
var fao40_20 = FAO.updateMask(gfc40_20);
//Map.addLayer(fao40_00, vis_40, 'Hansen GFC FAO 2000 >40%');
//Map.addLayer(fao40_20, vis_40_loss, 'Hansen GFC FAO 2020 >40%');

var fao_40_00 = fao40_00.visualize({
  bands: ['b1'],
  max: 1
});
Export.image.toDrive({
  image: fao_40_00,
  description: 'fao_40_00',
  scale: 30,
  maxPixels: 1e13,
  region: tropics
});

var fao_40_20 = fao40_20.visualize({
  bands: ['b1'],
  max: 1
});
Export.image.toDrive({
  image: fao_40_20,
  description: 'fao_40_20',
  scale: 30,
  maxPixels: 1e13,
  region: tropics
});
```

Statistical Analyses of Tropical Dry Forest Extent

Global, regions, Biodiversity Hotspots, countries, and islands level data on area of tropical dry forest extent based on climatic definitions and data sets were examined for a normal distribution using one-sample Shapiro-Wilk normality test for small samples (< 30) and Kolmogorov-Smirnov tests (> 30). Parametric (T-tests) and non-parametric (Wilcoxon rank sum test) tests were used to identify significant differences among climatic definitions and between Worldclim and CHELSA data sets at a global, regional (regions, Biodiversity Hotspots, countries), and local (island archipelagos) spatial scales.

Non-parametric and Paired T-tests

```
#### n < 50 (Ecos, Hotspots, Regions) ####
files <- list.files(getwd(), pattern = '.csv', full.names = F)

df <- read_csv("countries-stats.csv") %>%
  select(-Region) %>%
  data.frame()
df[is.na(df)] <- 0

## one-sample Shapiro-Wilk Normality Test
for (j in names(df)){
  sink("Shapiro-Wilk.txt", append = T)
  cat("=============================\n")
  cat(paste(j, "\n"))
  cat("=============================\n")

  mww <- capture.output(shapiro.test(df[[j]]))

  cat(mww, file = "Shapiro-Wilk.txt", sep = "\n", append = T)
  sink()
}

## Mann-Whitney-Wilcox Non-Parametric Test
def <- c("m","f","d","a")
ch <- "_ch"
wc <- "_wc"

for (j in unique(def)){
  sink("Mann-Whitney-Wilcox.txt", append = T)
  cat("=============================\n")
  cat(paste(j, ch, ', ', j, wc, sep = '', "\n"))
  cat("=============================\n")

  x <- df[[paste(j, ch, sep = '')]]
  y <- df[[paste(j, wc, sep = '')]]

  t <- capture.output(wilcox.test(x, y))

  cat(t,file="Mann-Whitney-Wilcox.txt", sep = "\n", append = T)
  sink()
}
```

Data Visualization

The final chunks of code were used to create visualizations of our analyses. These include density charts and box plots for statistical analysis and dozens of global, regional, and local maps of tropical dry forest extent and cover.

Statistical Charts

```
## Plots
df <- df %>%
  gather("m_ch", "f_ch", "d_ch", "a_ch",
         "m_wc", "f_wc", "d_wc", "a_wc",
         key = "text",
         value = "value") %>%
  mutate(value = round(as.numeric(value),0))

# Boxplot
ggplot(df, aes(df$text, df$value, fill = df$text)) +
  geom_boxplot() +
  theme_ipsum() +
  ylab("Extent (Km^2)") +
  xlab("Definition (Dataset)") +
  theme(
    axis.line = element_line(colour = "darkblue", size = 2, linetype = "solid"),
    axis.title.y = element_text(size = 16),
    axis.title.x = element_text(size = 16),
    legend.position = "none",
    panel.background = element_rect(fill = "ghostwhite"),
    panel.grid.major = element_line(size = 1 , linetype = "solid" , colour = "white"),
    panel.grid.minor = element_line(size = 1 , linetype = "solid" , colour = "white"))

# Density Plots
lab <- c("Murphy & Lugo (CHELSA)", "FAO (CHELSA)", "Dryflor (CHELSA)", "Aridity (CHELSA)",
         "Murphy & Lugo (Worldclim)", "FAO (Worldclim)", "Dryflor (Worldclim)", "Aridity (Worldclim)")
```

```r
df$text <- lab

ggplot(df, aes(df$value, group = df$text, fill = df$text)) +
  geom_density() +
  theme_ipsum() +
  xlab("Extent (Km^2)") +
  ylab("Density") +
  facet_wrap(~df$text, nrow = 2) +
  theme(
    axis.title.y = element_text(size = 16),
    axis.title.x = element_text(size = 16),
    panel.background = element_rect(fill = "ghostwhite"),
    panel.grid.major = element_line(size = 1 , linetype = "solid" , colour = "white"),
    panel.grid.minor = element_line(size = 1 , linetype = "solid" , colour = "white"),
    legend.position="none",
    panel.spacing = unit(0.1, "lines"),
    axis.ticks.x=element_blank())

# Stacked Density Plot
ggplot(df, aes(df$value, group = df$text, fill = df$text)) +
  geom_density(alpha = 0.4) +
  theme_ipsum() +
  xlab("Extent (Km^2)") +
  ylab("Density") +
  labs(fill = "Definition (Dataset)") +
  theme(
    axis.line = element_line(colour = "darkblue", size = 2, linetype = "solid"),
    axis.title.y = element_text(size = 16),
    axis.title.x = element_text(size = 16),
    legend.text = element_text(size = 12),
    legend.title = element_text(color = "darkblue", size = 16),
    legend.background = element_rect(fill = "white"),
    legend.position = c(0.8, 0.7),
    panel.background = element_rect(fill = "ghostwhite"),
    panel.grid.major = element_line(size = 1 , linetype = "solid" , colour = "white"),
    panel.grid.minor = element_line(size = 1 , linetype = "solid" , colour = "white"))
```

Tropical Dry Forest Maps

```r
#polygonize rasters into shapefile - ONLY RUN ONCE####
gdal_polygonizeR <- function(x, outshape = NULL, gdalformat = "ESRI Shapefile",
                             pypath = NULL, readpoly = T, quiet = T) {
  if (isTRUE(readpoly)) require(rgdal)
  if (is.null(pypath)) {
    pypath <- Sys.which("gdal_polygonize.py")
  }
  if (!file.exists(pypath)) stop("gdal_polygonize.py not on system!")
  owd <- getwd()
  on.exit(setwd(owd))
  setwd(dirname(pypath))
  if (!is.null(outshape)) {
    outshape <- sub("\\.shp$", "", outshape)
    f.exists <- file.exists(paste(outshape, c("shp", "shx", "dbf"), sep = "."))
    if (any(f.exists))
      stop(sprintf("File already exists: %s",
                   toString(paste(outshape, c("shp", "shx", "dbf"),
                                  sep = '.')[f.exists])), call. = F)
  } else outshape <- tempfile()
  if (is(x, "Raster")) {
    require(raster)
    writeRaster(x, {f <- tempfile(fileext = ".asc")})
    rastpath <- normalizePath(f)
  } else if (is.character(x)) {
    rastpath <- normalizePath(x)
  } else stop("'x' must be a file path (char str), or a raster object.")
  system2("python", args=(sprintf('"%1$s" "%2$s" -f "%3$s" "%4$s.shp"',
                                  pypath, rastpath, gdalformat, outshape)))
  if (isTRUE(readpoly)) {
    shp <- readOGR(dirname(outshape), layer = basename(outshape), verbose=!quiet)
    return(shp)
  }
  return(NULL)
}

land <- gdal_polygonizeR(file.path(cwd, "data/baselayers/land_tropics.tif"), readpoly = T) %>%
  gBuffer(byid = T, width = 0) #fix geoms
#tropics
poly <- readWKT("POLYGON((
                -180.0 -30.0,
                180.0 -30.0,
                180.0 30.0,
                -180.0 30.0,
                -180.0 -30.0
))", p4s = CRS("+proj=longlat +datum=WGS84"))
land <- crop_shape(land, poly)
#rasters
climate <- list.files("data/climate/proc_clim/", pattern = ".tif", full.names = T)
#Run the polygonize function over each raster called in the variable climate

#mapping####
tmap_mode("plot")
abc <- c("a.", "b.", "c.", "d.", "e.", "f.", "g.", "h.")




#PLOTS####
tdf_plots <- tm_shape(land) + #basemap
  tm_fill(col = "#FFFFFF") +

  tm_shape(countries) + #countries
  tm_borders(col = "#CCCCCC") +
```

```r
  tm_shape(ecoregions) + #ecoregions
  tm_fill(col = "#758000",
          alpha = 0.8) +

  tm_shape(plots) + #TDF plots
  tm_dots(size = 0.1,
          col = "#CC0000",
          legend.z = 0) +

  tm_add_legend("symbol", col = "#758000",
                shape = 15, size = 0.75, #sizing helps stack symbols
                alpha = 0.8, labels = "WWF TSBF Ecoregions") +
  tm_add_legend("symbol", col = "#CC0000",
                shape = 16, size = 0.75,
                labels = "Tropical Dry Forest Plots") +

  tm_layout(bg.color = "#A6CEE3", #ocean
            outer.margins = c(0, 0, 0, 0),
            legend.position = c(0.01, 0.05), #coords (left, bottom)
            legend.text.size = 0.5, #sizing helps stack text
            legend.width = -1, #prevents any auto re-sizing
            fontfamily = "Times")


tmap_save(tdf_plots, "maps/tdf_plots.png",
          width = 7, units = "in", asp = 0)




#AI####
tdf_wc_65 <- tm_shape(land) + #basemap
  tm_fill(col = "#FFFFFF") +

  tm_shape(countries) + #countries
  tm_borders(col = "#CCCCCC") +

  tm_shape(wc_65) + #Worldclim AI
  tm_fill(col = "#33638D") +

  tm_add_legend("symbol", col = "#33638D", title = "Worldclim v2",
                shape = 15, size = 0.75, #sizing helps stack symbols
                alpha = 0.8, labels = "AI <= 0.65") +

  tm_layout(bg.color = "#A6CEE3", #ocean
            main.title = "a.",
            main.title.position = 0,
            outer.margins = c(0, 0, 0, 0),
            legend.position = c(0.01, 0.05), #coords (left, bottom)
            legend.title.size = 0.8,
            legend.text.size = 0.5, #sizing helps stack text
            legend.width = -1, #prevents any auto re-sizing
            fontfamily = "Times")


tdf_ch_65 <- tm_shape(land) + #basemap
  tm_fill(col = "#FFFFFF") +

  tm_shape(countries) + #countries
  tm_borders(col = "#CCCCCC") +

  tm_shape(ch_65) + #CHLESA AI
  tm_fill(col = "#33638D") +

  tm_add_legend("symbol", col = "#33638D", title = "CHELSA",
                shape = 15, size = 0.75, #sizing helps stack symbols
                alpha = 0.8, labels = "AI <= 0.65") +

  tm_layout(bg.color = "#A6CEE3", #ocean
            main.title = "b.",
            main.title.position = 0,
            outer.margins = c(0, 0, 0, 0),
            legend.position = c(0.01, 0.05), #coords (left, bottom)
            legend.title.size = 0.8,
            legend.text.size = 0.5, #sizing helps stack text
            legend.width = -1, #prevents any auto re-sizing
            fontfamily = "Times")


tdf_overlap_ai <- tm_shape(land) + #basemap
  tm_fill(col = "#FFFFFF") +

  tm_shape(countries) + #countries
  tm_borders(col = "#CCCCCC") +

  tm_shape(raster(climate[8])) + #Consensus AI
  tm_raster(palette = c("#3DBC74", "#33638D"),
            legend.show = F) +

  tm_add_legend("symbol", col = c("#3DBC74", "#33638D"), title = "AI Comparison",
                shape = 15, size = 0.75, #sizing helps stack symbols
                alpha = 0.8, labels = c("No Consensus", "Consensus")) +

  tm_layout(bg.color = "#A6CEE3", #ocean
            main.title = "c.",
            main.title.position = 0,
            outer.margins = c(0, 0, 0, 0),
            legend.position = c(0.01, 0.05), #coords (left, bottom)
            legend.title.size = 0.8,
            legend.text.size = 0.5, #sizing helps stack text
            legend.width = -1, #prevents any auto re-sizing
            fontfamily = "Times")


list_ai <- list(tdf_wc_65, tdf_ch_65, tdf_overlap_ai) #list ai maps
tdf_ai <- tmap_arrange(list_ai, ncol = 1, nrow = 3,
                       asp = NA, outer.margins = NULL) #stack/arrange maps vertically
```

```r
tmap_save(tdf_ai, "maps/tdf_ai.png", width = 7, units = "in")



#WORLDCLIM####
tdf_wc_ml <- tm_shape(land) + #basemap
  tm_fill(col = "#FFFFFF") +

  tm_shape(countries) + #countries
  tm_borders(col = "#CCCCCC") +

  tm_shape(ml_wc) + #Worldclim Murphy and Lugo
  tm_fill(col = "#33638D") +

  tm_add_legend("symbol", col = "#33638D", title = "Worldclim v2",
                shape = 15, size = 0.75, #sizing helps stack symbols
                alpha = 0.8, labels = "Murphy and Lugo") +

  tm_layout(bg.color = "#A6CEE3", #ocean
            main.title = "a.",
            main.title.position = 0,
            outer.margins = c(0, 0, 0, 0),
            legend.position = c(0.01, 0.05), #coords (left, bottom)
            legend.title.size = 0.8,
            legend.text.size = 0.5, #sizing helps stack text
            legend.width = -1, #prevents any auto re-sizing
            fontfamily = "Times")


tdf_wc_fao <- tm_shape(land) + #basemap
  tm_fill(col = "#FFFFFF") +

  tm_shape(countries) + #countries
  tm_borders(col = "#CCCCCC") +

  tm_shape(fao_wc) + #Worldclim FAO
  tm_fill(col = "#33638D") +

  tm_add_legend("symbol", col = "#33638D", title = "Worldclim v2",
                shape = 15, size = 0.75, #sizing helps stack symbols
                alpha = 0.8, labels = "FAO") +

  tm_layout(bg.color = "#A6CEE3", #ocean
            main.title = "b.",
            main.title.position = 0,
            outer.margins = c(0, 0, 0, 0),
            legend.position = c(0.01, 0.05), #coords (left, bottom)
            legend.title.size = 0.8,
            legend.text.size = 0.5, #sizing helps stack text
            legend.width = -1, #prevents any auto re-sizing
            fontfamily = "Times")


tdf_wc_dry <- tm_shape(land) + #basemap
  tm_fill(col = "#FFFFFF") +

  tm_shape(countries) + #countries
  tm_borders(col = "#CCCCCC") +

  tm_shape(dry_wc) + #Worldclim Dryflor
  tm_fill(col = "#33638D") +

  tm_add_legend("symbol", col = "#33638D", title = "Worldclim v2",
                shape = 15, size = 0.75, #sizing helps stack symbols
                alpha = 0.8, labels = "Dryflor") +

  tm_layout(bg.color = "#A6CEE3", #ocean
            main.title = "c.",
            main.title.position = 0,
            outer.margins = c(0, 0, 0, 0),
            legend.position = c(0.01, 0.05), #coords (left, bottom)
            legend.title.size = 0.8,
            legend.text.size = 0.5, #sizing helps stack text
            legend.width = -1, #prevents any auto re-sizing
            fontfamily = "Times")


tdf_overlap_wc <- tm_shape(land) + #basemap
  tm_fill(col = "#FFFFFF") +

  tm_shape(countries) + #countries
  tm_borders(col = "#CCCCCC") +

  tm_shape(raster(climate[10])) + #Consensus WC
  tm_raster(palette = c("#33638D", "#3DBC74", "#CAE11F"),
            legend.show = F) +

  tm_add_legend("symbol", col = c("#33638D", "#3DBC74", "#CAE11F"), title = "Worldclim Comparison",
                shape = 15, size = 0.75, #sizing helps stack symbols
                alpha = 0.8, labels = c("No Consensus", "Consensus Among 2", "Consensus Among All")) +

  tm_layout(bg.color = "#A6CEE3", #ocean
            main.title = "d.",
            main.title.position = 0,
            outer.margins = c(0, 0, 0, 0),
            legend.position = c(0.01, 0.05), #coords (left, bottom)
            legend.title.size = 0.8,
            legend.text.size = 0.5, #sizing helps stack text
            legend.width = -1, #prevents any auto re-sizing
            fontfamily = "Times")


list_wc <- list(tdf_wc_ml, tdf_wc_fao, tdf_wc_dry, tdf_overlap_wc) #list wc maps
tdf_wc <- tmap_arrange(list_wc, ncol = 1, nrow = 4,
```

```r
                        asp = NA, outer.margins = NULL) #stack/arrange maps vertically


tmap_save(tdf_wc, "maps/tdf_wc.png", width = 7, units = "in")




#CHELSA####
tdf_ch_ml <- tm_shape(land) + #basemap
  tm_fill(col = "#FFFFFF") +

  tm_shape(countries) + #countries
  tm_borders(col = "#CCCCCC") +

  tm_shape(ml_ch) + #CHELSA Murphy and Lugo
  tm_fill(col = "#33638D") +

  tm_add_legend("symbol", col = "#33638D", title = "CHELSA",
                shape = 15, size = 0.75, #sizing helps stack symbols
                alpha = 0.8, labels = "Murphy and Lugo") +

  tm_layout(bg.color = "#A6CEE3", #ocean
            main.title = "a.",
            main.title.position = 0,
            outer.margins = c(0, 0, 0, 0),
            legend.position = c(0.01, 0.05), #coords (left, bottom)
            legend.title.size = 0.8,
            legend.text.size = 0.5, #sizing helps stack text
            legend.width = -1, #prevents any auto re-sizing
            fontfamily = "Times")


tdf_ch_fao <- tm_shape(land) + #basemap
  tm_fill(col = "#FFFFFF") +

  tm_shape(countries) + #countries
  tm_borders(col = "#CCCCCC") +

  tm_shape(fao_ch) + #CHELSA FAO
  tm_fill(col = "#33638D") +

  tm_add_legend("symbol", col = "#33638D", title = "CHELSA",
                shape = 15, size = 0.75, #sizing helps stack symbols
                alpha = 0.8, labels = "FAO") +

  tm_layout(bg.color = "#A6CEE3", #ocean
            main.title = "b.",
            main.title.position = 0,
            outer.margins = c(0, 0, 0, 0),
            legend.position = c(0.01, 0.05), #coords (left, bottom)
            legend.title.size = 0.8,
            legend.text.size = 0.5, #sizing helps stack text
            legend.width = -1, #prevents any auto re-sizing
            fontfamily = "Times")


tdf_ch_dry <- tm_shape(land) + #basemap
  tm_fill(col = "#FFFFFF") +

  tm_shape(countries) + #countries
  tm_borders(col = "#CCCCCC") +

  tm_shape(dry_ch) + #CHELSA Dryflor
  tm_fill(col = "#33638D") +

  tm_add_legend("symbol", col = "#33638D", title = "CHELSA",
                shape = 15, size = 0.75, #sizing helps stack symbols
                alpha = 0.8, labels = "Dryflor") +

  tm_layout(bg.color = "#A6CEE3", #ocean
            main.title = "c.",
            main.title.position = 0,
            outer.margins = c(0, 0, 0, 0),
            legend.position = c(0.01, 0.05), #coords (left, bottom)
            legend.title.size = 0.8,
            legend.text.size = 0.5, #sizing helps stack text
            legend.width = -1, #prevents any auto re-sizing
            fontfamily = "Times")


tdf_overlap_ch <- tm_shape(land) + #basemap
  tm_fill(col = "#FFFFFF") +

  tm_shape(countries) + #countries
  tm_borders(col = "#CCCCCC") +

  tm_shape(raster(climate[9])) + #Consensus CH
  tm_raster(palette = c("#33638D", "#3DBC74", "#CAE11F"),
            legend.show = F) +

  tm_add_legend("symbol", col = c("#33638D", "#3DBC74", "#CAE11F"), title = "CHELSA Comparison",
                shape = 15, size = 0.75, #sizing helps stack symbols
                alpha = 0.8, labels = c("No Consensus", "Consensus Among 2", "Consensus Among All")) +

  tm_layout(bg.color = "#A6CEE3", #ocean
            main.title = "d.",
            main.title.position = 0,
            outer.margins = c(0, 0, 0, 0),
            legend.position = c(0.01, 0.05), #coords (left, bottom)
            legend.title.size = 0.8,
            legend.text.size = 0.5, #sizing helps stack text
            legend.width = -1, #prevents any auto re-sizing
            fontfamily = "Times")


list_ch <- list(tdf_ch_ml, tdf_ch_fao, tdf_ch_dry, tdf_overlap_ch) #list ch maps
```

```
tdf_ch <- tmap_arrange(list_ch, ncol = 1, nrow = 4,
                       asp = NA, outer.margins = NULL) #stack/arrange maps vertically


tmap_save(tdf_ch, "maps/tdf_ch.png", width = 7, units = "in")

#Following plotting code can be edited for data set and region...
#Subcontinents####
for (i in 1:5){
  subcon <- readOGR(subcons[i]) %>% gBuffer(byid = T, width = 0)

  if (i == 3){


    PACsubcon <- spTransform(subcon, CRSobj = "+proj=merc +lon_0=150 +k=1 +x_0=0 +y_0=0 +datum=WGS84 +units=m +no_defs") %>% gBuffer(byid = T, width = 0)
    PACcountries <- spTransform(countries, CRSobj = "+proj=merc +lon_0=150 +k=1 +x_0=0 +y_0=0 +datum=WGS84 +units=m +no_defs") %>% gBuffer(byid = T, width = 0)
    PACplots <- spTransform(plots, CRSobj = "+proj=merc +lon_0=150 +k=1 +x_0=0 +y_0=0 +datum=WGS84 +units=m +no_defs ")

    PACml_ch <- spTransform(ml_ch, CRSobj = "+proj=merc +lon_0=150 +k=1 +x_0=0 +y_0=0 +datum=WGS84 +units=m +no_defs ") %>% gBuffer(byid = T, width = 0)
    PACfao_ch <- spTransform(fao_ch, CRSobj = "+proj=merc +lon_0=150 +k=1 +x_0=0 +y_0=0 +datum=WGS84 +units=m +no_defs ") %>% gBuffer(byid = T, width = 0)
    PACdry_ch <- spTransform(dry_ch, CRSobj = "+proj=merc +lon_0=150 +k=1 +x_0=0 +y_0=0 +datum=WGS84 +units=m +no_defs ") %>% gBuffer(byid = T, width = 0)


    PACsubcon_ml_ch <- tm_shape(PACsubcon) + #basemap
      tm_fill(col = "#FFFFFF") +
      tm_shape(raster::crop(PACcountries, PACsubcon)) + #countries
      tm_borders(col = "#CCCCCC") +
      tm_shape(raster::crop(PACml_ch, PACsubcon)) + #ML CHELSA
      tm_fill(col = "#33638D") +
      tm_shape(raster::crop(PACplots, PACsubcon)) + #TDF plots
      tm_symbols(size = 0.025, shape = 21, col = "#CC0000",
                 border.lwd = 0.5, border.col = "#CCCCCC") +
      tm_add_legend("symbol", col = "#CC0000",
                    shape = 16, size = 0.75,
                    labels = "Tropical Dry Forest Plots") +
      tm_add_legend("symbol", col = "#33638D",
                    shape = 15, size = 0.75, #sizing helps stack symbols
                    alpha = 0.8, labels = "Murphy and Lugo, CHELSA") +
      tm_layout(bg.color = "#A6CEE3", #ocean
                main.title = abc[i],
                main.title.position = 0,
                inner.margins = c(0.3, 0, 0, 0),
                legend.position = c(0.01, 0.05), #coords (left, bottom)
                legend.text.size = 0.5, #sizing helps stack text
                legend.width = -1, #prevents any auto re-sizing
                fontfamily = "Times")


    PACsubcon_fao_ch <- tm_shape(PACsubcon) + #basemap
      tm_fill(col = "#FFFFFF") +
      tm_shape(raster::crop(PACcountries, PACsubcon)) + #countries
      tm_borders(col = "#CCCCCC") +
      tm_shape(raster::crop(PACfao_ch, PACsubcon)) + #FAO CHELSA
      tm_fill(col = "#33638D") +
      tm_shape(raster::crop(PACplots, PACsubcon)) + #TDF plots
      tm_symbols(size = 0.025, shape = 21, col = "#CC0000",
                 border.lwd = 0.5, border.col = "#CCCCCC") +
      tm_add_legend("symbol", col = "#CC0000",
                    shape = 16, size = 0.75,
                    labels = "Tropical Dry Forest Plots") +
      tm_add_legend("symbol", col = "#33638D",
                    shape = 15, size = 0.75, #sizing helps stack symbols
                    alpha = 0.8, labels = "FAO, CHELSA") +
      tm_layout(bg.color = "#A6CEE3", #ocean
                main.title = " ",
                main.title.position = 0,
                inner.margins = c(0.3, 0, 0, 0),
                legend.position = c(0.01, 0.05), #coords (left, bottom)
                legend.text.size = 0.5, #sizing helps stack text
                legend.width = -1, #prevents any auto re-sizing
                fontfamily = "Times")


    PACsubcon_dry_ch <- tm_shape(PACsubcon) + #basemap
      tm_fill(col = "#FFFFFF") +
      tm_shape(raster::crop(PACcountries, PACsubcon)) + #countries
      tm_borders(col = "#CCCCCC") +
      tm_shape(raster::crop(PACdry_ch, PACsubcon)) + #Dryflor CHELSA
      tm_fill(col = "#33638D") +
      tm_shape(raster::crop(PACplots, PACsubcon)) + #TDF plots
      tm_symbols(size = 0.025, shape = 21, col = "#CC0000",
                 border.lwd = 0.5, border.col = "#CCCCCC") +
      tm_add_legend("symbol", col = "#CC0000",
                    shape = 16, size = 0.75,
                    labels = "Tropical Dry Forest Plots") +
      tm_add_legend("symbol", col = "#33638D",
                    shape = 15, size = 0.75, #sizing helps stack symbols
                    alpha = 0.8, labels = "Dryflor, CHELSA") +
      tm_layout(bg.color = "#A6CEE3", #ocean
                main.title = " ",
                main.title.position = 0,
                inner.margins = c(0.3, 0, 0, 0),
                legend.position = c(0.01, 0.05), #coords (left, bottom)
                legend.text.size = 0.5, #sizing helps stack text
                legend.width = -1, #prevents any auto re-sizing
                fontfamily = "Times")


    #inset of Hawaii
    hawaii <- readOGR(archs[3]) %>%
      spTransform(CRSobj = "+proj=merc +lon_0=150 +k=1 +x_0=0 +y_0=0 +datum=WGS84 +units=m +no_defs") %>%
      gBuffer(byid = T, width = 0)


    PACH_ml_ch <-  tm_shape(hawaii) +
      tm_fill(col = "#FFFFFF") +
      tm_shape(raster::crop(PACcountries, hawaii)) + #countries
```

```r
    tm_borders(col = "#CCCCCC") +
    tm_shape(raster::crop(PACml_ch, hawaii)) +
    tm_fill(col = "#33638D") +
    tm_shape(raster::crop(PACplots, hawaii)) +
    tm_symbols(size = 0.01, shape = 21, col = "#CC0000",
               border.lwd = 0.5, border.col = "#CCCCCC") +
    tm_layout(bg.color = "#A6CEE3")


  PACH_fao_ch <- tm_shape(hawaii) +
    tm_fill(col = "#FFFFFF") +
    tm_shape(raster::crop(PACcountries, hawaii)) + #countries
    tm_borders(col = "#CCCCCC") +
    tm_shape(raster::crop(PACfao_ch, hawaii)) +
    tm_fill(col = "#33638D") +
    tm_shape(raster::crop(PACplots, hawaii)) +
    tm_symbols(size = 0.01, shape = 21, col = "#CC0000",
               border.lwd = 0.5, border.col = "#CCCCCC") +
    tm_layout(bg.color = "#A6CEE3")


  PACH_dry_ch <- tm_shape(hawaii) +
    tm_fill(col = "#FFFFFF") +
    tm_shape(raster::crop(PACcountries, hawaii)) + #countries
    tm_borders(col = "#CCCCCC") +
    tm_shape(raster::crop(PACdry_ch, hawaii)) +
    tm_fill(col = "#33638D") +
    tm_shape(raster::crop(PACplots, hawaii)) +
    tm_symbols(size = 0.01, shape = 21, col = "#CC0000",
               border.lwd = 0.5, border.col = "#CCCCCC") +
    tm_layout(bg.color = "#A6CEE3")


  #save maps with insets
  tmap_save(PACsubcon_ml_ch, paste("maps/pac_ch_1.png", sep = ""),
            insets_tm = PACH_ml_ch, insets_vp = viewport(x = 0.75, y = 0.65, width = 0.4, height = 0.4),
            width = 7/3, height = 2, units = "in", asp = 0)
  tmap_save(PACsubcon_fao_ch, paste("maps/pac_ch_2.png", sep = ""),
            insets_tm = PACH_fao_ch, insets_vp = viewport(x = 0.75, y = 0.65, width = 0.4, height = 0.4),
            width = 7/3, height = 2, units = "in", asp = 0)
  tmap_save(PACsubcon_dry_ch, paste("maps/pac_ch_3.png", sep = ""),
            insets_tm = PACH_dry_ch, insets_vp = viewport(x = 0.75, y = 0.65, width = 0.4, height = 0.4),
            width = 7/3, height = 2, units = "in", asp = 0)


  #call maps back in and save
  pnglist = lapply(list.files("maps", pattern = "pac_ch_*", full.names = T), png::readPNG)
  groblist = lapply(pnglist, grid::rasterGrob)
  pac_ch <- gridExtra::grid.arrange(grobs = groblist, nrow = 1)
  ggsave(paste("maps/tdf_fig6_", i, ".png", sep = ""),
         pac_ch, width = 7, height = 2, units = "in")


} else {


  subcon_ml_ch <- tm_shape(subcon) + #basemap
    tm_fill(col = "#FFFFFF") +
    tm_shape(raster::crop(countries, subcon)) + #countries
    tm_borders(col = "#CCCCCC") +
    tm_shape(raster::crop(ml_ch, subcon)) + #ML CHELSA
    tm_fill(col = "#33638D") +
    tm_shape(raster::crop(plots, subcon)) + #TDF plots
    tm_symbols(size = 0.025, shape = 21, col = "#CC0000",
               border.lwd = 0.5, border.col = "#CCCCCC") +
    tm_add_legend("symbol", col = "#CC0000",
                  shape = 16, size = 0.75,
                  labels = "Tropical Dry Forest Plots") +
    tm_add_legend("symbol", col = "#33638D",
                  shape = 15, size = 0.75, #sizing helps stack symbols
                  alpha = 0.8, labels = "Murphy and Lugo, CHELSA") +
    tm_layout(bg.color = "#A6CEE3", #ocean
              main.title = abc[i],
              main.title.position = 0,
              inner.margins = c(0.3, 0, 0, 0),
              legend.position = c(0.01, 0.05), #coords (left, bottom)
              legend.text.size = 0.5, #sizing helps stack text
              legend.width = -1, #prevents any auto re-sizing
              fontfamily = "Times")


  subcon_fao_ch <- tm_shape(subcon) + #basemap
    tm_fill(col = "#FFFFFF") +
    tm_shape(raster::crop(countries, subcon)) + #countries
    tm_borders(col = "#CCCCCC") +
    tm_shape(raster::crop(fao_ch, subcon)) + #FAO CHELSA
    tm_fill(col = "#33638D") +
    tm_shape(raster::crop(plots, subcon)) + #TDF plots
    tm_symbols(size = 0.025, shape = 21, col = "#CC0000",
               border.lwd = 0.5, border.col = "#CCCCCC") +
    tm_add_legend("symbol", col = "#CC0000",
                  shape = 16, size = 0.75,
                  labels = "Tropical Dry Forest Plots") +
    tm_add_legend("symbol", col = "#33638D",
                  shape = 15, size = 0.75, #sizing helps stack symbols
                  alpha = 0.8, labels = "FAO, CHELSA") +
    tm_layout(bg.color = "#A6CEE3", #ocean
              main.title = " ",
              main.title.position = 0,
              inner.margins = c(0.3, 0, 0, 0),
              legend.position = c(0.01, 0.05), #coords (left, bottom)
              legend.text.size = 0.5, #sizing helps stack text
              legend.width = -1, #prevents any auto re-sizing
              fontfamily = "Times")


  subcon_dry_ch <- tm_shape(subcon) + #basemap
```

```
      tm_fill(col = "#FFFFFF") +
      tm_shape(raster::crop(countries, subcon)) + #countries
      tm_borders(col = "#CCCCCC") +
      tm_shape(raster::crop(dry_ch, subcon)) + #Dryflor CHELSA
      tm_fill(col = "#33638D") +
      tm_shape(raster::crop(plots, subcon)) + #TDF plots
      tm_symbols(size = 0.025, shape = 21, col = "#CC0000",
                 border.lwd = 0.5, border.col = "#CCCCCC") +
      tm_add_legend("symbol", col = "#CC0000",
                    shape = 16, size = 0.75,
                    labels = "Tropical Dry Forest Plots") +
      tm_add_legend("symbol", col = "#33638D",
                    shape = 15, size = 0.75, #sizing helps stack symbols
                    alpha = 0.8, labels = "Dryflor, CHELSA") +
      tm_layout(bg.color = "#A6CEE3", #ocean
                main.title = " ",
                main.title.position = 0,
                inner.margins = c(0.3, 0, 0, 0),
                legend.position = c(0.01, 0.05), #coords (left, bottom)
                legend.text.size = 0.5, #sizing helps stack text
                legend.width = -1, #prevents any auto re-sizing
                fontfamily = "Times")


    #save maps with insets
    tmap_save(subcon_ml_ch, paste("maps/sub_ch_1.png", sep = ""),
              width = 7/3, height = 2, units = "in", asp = 0)
    tmap_save(subcon_fao_ch, paste("maps/sub_ch_2.png", sep = ""),
              width = 7/3, height = 2, units = "in", asp = 0)
    tmap_save(subcon_dry_ch, paste("maps/sub_ch_3.png", sep = ""),
              width = 7/3, height = 2, units = "in", asp = 0)


    #call maps back in and save
    pnglist = lapply(list.files("maps", pattern = "sub_ch_*", full.names = T), png::readPNG)
    groblist = lapply(pnglist, grid::rasterGrob)
    sub_ch <- gridExtra::grid.arrange(grobs = groblist, nrow = 1)
    ggsave(paste("maps/tdf_fig6_", i, ".png", sep = ""),
           sub_ch, width = 7, height = 2, units = "in")


  }
} #CHELSA


for (i in 1:5){
  subcon <- readOGR(subcons[i]) %>% gBuffer(byid = T, width = 0)

  if (i == 3){


    PACsubcon <- spTransform(subcon, CRSobj = "+proj=merc +lon_0=150 +k=1 +x_0=0 +y_0=0 +datum=WGS84 +units=m +no_defs") %>% gBuffer(byid = T, width = 0)
    PACcountries <- spTransform(countries, CRSobj = "+proj=merc +lon_0=150 +k=1 +x_0=0 +y_0=0 +datum=WGS84 +units=m +no_defs") %>% gBuffer(byid = T, width = 0)
    PACplots <- spTransform(plots, CRSobj = "+proj=merc +lon_0=150 +k=1 +x_0=0 +y_0=0 +datum=WGS84 +units=m +no_defs ")

    PACml_wc <- spTransform(ml_ch, CRSobj = "+proj=merc +lon_0=150 +k=1 +x_0=0 +y_0=0 +datum=WGS84 +units=m +no_defs ") %>% gBuffer(byid = T, width = 0)
    PACfao_wc <- spTransform(fao_ch, CRSobj = "+proj=merc +lon_0=150 +k=1 +x_0=0 +y_0=0 +datum=WGS84 +units=m +no_defs ") %>% gBuffer(byid = T, width = 0)
    PACdry_wc <- spTransform(dry_ch, CRSobj = "+proj=merc +lon_0=150 +k=1 +x_0=0 +y_0=0 +datum=WGS84 +units=m +no_defs ") %>% gBuffer(byid = T, width = 0)


    PACsubcon_ml_wc <- tm_shape(PACsubcon) + #basemap
      tm_fill(col = "#FFFFFF") +
      tm_shape(raster::crop(PACcountries, PACsubcon)) + #countries
      tm_borders(col = "#CCCCCC") +
      tm_shape(raster::crop(PACml_wc, PACsubcon)) + #ML Worldclim
      tm_fill(col = "#33638D") +
      tm_shape(raster::crop(PACplots, PACsubcon)) + #TDF plots
      tm_symbols(size = 0.025, shape = 21, col = "#CC0000",
                 border.lwd = 0.5, border.col = "#CCCCCC") +
      tm_add_legend("symbol", col = "#CC0000",
                    shape = 16, size = 0.75,
                    labels = "Tropical Dry Forest Plots") +
      tm_add_legend("symbol", col = "#33638D",
                    shape = 15, size = 0.75, #sizing helps stack symbols
                    alpha = 0.8, labels = "Murphy and Lugo, Worldclim") +
      tm_layout(bg.color = "#A6CEE3", #ocean
                main.title = abc[i],
                main.title.position = 0,
                inner.margins = c(0.3, 0, 0, 0),
                legend.position = c(0.01, 0.05), #coords (left, bottom)
                legend.text.size = 0.5, #sizing helps stack text
                legend.width = -1, #prevents any auto re-sizing
                fontfamily = "Times")


    PACsubcon_fao_wc <- tm_shape(PACsubcon) + #basemap
      tm_fill(col = "#FFFFFF") +
      tm_shape(raster::crop(PACcountries, PACsubcon)) + #countries
      tm_borders(col = "#CCCCCC") +
      tm_shape(raster::crop(PACfao_wc, PACsubcon)) + #FAO Worldclim
      tm_fill(col = "#33638D") +
      tm_shape(raster::crop(PACplots, PACsubcon)) + #TDF plots
      tm_symbols(size = 0.025, shape = 21, col = "#CC0000",
                 border.lwd = 0.5, border.col = "#CCCCCC") +
      tm_add_legend("symbol", col = "#CC0000",
                    shape = 16, size = 0.75,
                    labels = "Tropical Dry Forest Plots") +
      tm_add_legend("symbol", col = "#33638D",
                    shape = 15, size = 0.75, #sizing helps stack symbols
                    alpha = 0.8, labels = "FAO, Worldclim") +
      tm_layout(bg.color = "#A6CEE3", #ocean
                main.title = " ",
                main.title.position = 0,
                inner.margins = c(0.3, 0, 0, 0),
                legend.position = c(0.01, 0.05), #coords (left, bottom)
                legend.text.size = 0.5, #sizing helps stack text
                legend.width = -1, #prevents any auto re-sizing
```

```r
              fontfamily = "Times")


  PACsubcon_dry_wc <- tm_shape(PACsubcon) + #basemap
    tm_fill(col = "#FFFFFF") +
    tm_shape(raster::crop(PACcountries, PACsubcon)) + #countries
    tm_borders(col = "#CCCCCC") +
    tm_shape(raster::crop(PACdry_wc, PACsubcon)) + #Dryflor Worldclim
    tm_fill(col = "#33638D") +
    tm_shape(raster::crop(PACplots, PACsubcon)) + #TDF plots
    tm_symbols(size = 0.025, shape = 21, col = "#CC0000",
               border.lwd = 0.5, border.col = "#CCCCCC") +
    tm_add_legend("symbol", col = "#CC0000",
                  shape = 16, size = 0.75,
                  labels = "Tropical Dry Forest Plots") +
    tm_add_legend("symbol", col = "#33638D",
                  shape = 15, size = 0.75, #sizing helps stack symbols
                  alpha = 0.8, labels = "Dryflor, Wordclim") +
    tm_layout(bg.color = "#A6CEE3", #ocean
              main.title = " ",
              main.title.position = 0,
              inner.margins = c(0.3, 0, 0, 0),
              legend.position = c(0.01, 0.05), #coords (left, bottom)
              legend.text.size = 0.5, #sizing helps stack text
              legend.width = -1, #prevents any auto re-sizing
              fontfamily = "Times")


  #inset of Hawaii
  hawaii <- readOGR(archs[3]) %>%
    spTransform(CRSobj = "+proj=merc +lon_0=150 +k=1 +x_0=0 +y_0=0 +datum=WGS84 +units=m +no_defs") %>%
    gBuffer(byid = T, width = 0)


  PACH_ml_wc <-  tm_shape(hawaii) +
    tm_fill(col = "#FFFFFF") +
    tm_shape(raster::crop(PACcountries, hawaii)) + #countries
    tm_borders(col = "#CCCCCC") +
    tm_shape(raster::crop(PACml_wc, hawaii)) +
    tm_fill(col = "#33638D") +
    tm_shape(raster::crop(PACplots, hawaii)) +
    tm_symbols(size = 0.01, shape = 21, col = "#CC0000",
               border.lwd = 0.5, border.col = "#CCCCCC") +
    tm_layout(bg.color = "#A6CEE3")


  PACH_fao_wc <- tm_shape(hawaii) +
    tm_fill(col = "#FFFFFF") +
    tm_shape(raster::crop(PACcountries, hawaii)) + #countries
    tm_borders(col = "#CCCCCC") +
    tm_shape(raster::crop(PACfao_wc, hawaii)) +
    tm_fill(col = "#33638D") +
    tm_shape(raster::crop(PACplots, hawaii)) +
    tm_symbols(size = 0.01, shape = 21, col = "#CC0000",
               border.lwd = 0.5, border.col = "#CCCCCC") +
    tm_layout(bg.color = "#A6CEE3")


  PACH_dry_wc <- tm_shape(hawaii) +
    tm_fill(col = "#FFFFFF") +
    tm_shape(raster::crop(PACcountries, hawaii)) + #countries
    tm_borders(col = "#CCCCCC") +
    tm_shape(raster::crop(PACdry_wc, hawaii)) +
    tm_fill(col = "#33638D") +
    tm_shape(raster::crop(PACplots, hawaii)) +
    tm_symbols(size = 0.01, shape = 21, col = "#CC0000",
               border.lwd = 0.5, border.col = "#CCCCCC") +
    tm_layout(bg.color = "#A6CEE3")


  #save maps with insets
  tmap_save(PACsubcon_ml_wc, paste("maps/pac_wc_1.png", sep = ""),
            insets_tm = PACH_ml_wc, insets_vp = viewport(x = 0.75, y = 0.65, width = 0.4, height = 0.4),
            width = 7/3, height = 2, units = "in", asp = 0)
  tmap_save(PACsubcon_fao_wc, paste("maps/pac_wc_2.png", sep = ""),
            insets_tm = PACH_fao_wc, insets_vp = viewport(x = 0.75, y = 0.65, width = 0.4, height = 0.4),
            width = 7/3, height = 2, units = "in", asp = 0)
  tmap_save(PACsubcon_dry_wc, paste("maps/pac_wc_3.png", sep = ""),
            insets_tm = PACH_dry_wc, insets_vp = viewport(x = 0.75, y = 0.65, width = 0.4, height = 0.4),
            width = 7/3, height = 2, units = "in", asp = 0)


  #call maps back in and save
  pnglist = lapply(list.files("maps", pattern = "pac_wc_*", full.names = T), png::readPNG)
  groblist = lapply(pnglist, grid::rasterGrob)
  pac_wc <- gridExtra::grid.arrange(grobs = groblist, nrow = 1)
  ggsave(paste("maps/tdf_fig7_", i, ".png", sep = ""),
         pac_wc, width = 7, height = 2, units = "in")


} else {


  subcon_ml_wc <- tm_shape(subcon) + #basemap
    tm_fill(col = "#FFFFFF") +
    tm_shape(raster::crop(countries, subcon)) + #countries
    tm_borders(col = "#CCCCCC") +
    tm_shape(raster::crop(ml_wc, subcon)) + #ML Worldclim
    tm_fill(col = "#33638D") +
    tm_shape(raster::crop(plots, subcon)) + #TDF plots
    tm_symbols(size = 0.025, shape = 21, col = "#CC0000",
               border.lwd = 0.5, border.col = "#CCCCCC") +
    tm_add_legend("symbol", col = "#CC0000",
                  shape = 16, size = 0.75,
                  labels = "Tropical Dry Forest Plots") +
    tm_add_legend("symbol", col = "#33638D",
                  shape = 15, size = 0.75, #sizing helps stack symbols
```

```r
                alpha = 0.8, labels = "Murphy and Lugo, Worldclim") +
    tm_layout(bg.color = "#A6CEE3", #ocean
              main.title = abc[i],
              main.title.position = 0,
              inner.margins = c(0.3, 0, 0, 0),
              legend.position = c(0.01, 0.05), #coords (left, bottom)
              legend.text.size = 0.5, #sizing helps stack text
              legend.width = -1, #prevents any auto re-sizing
              fontfamily = "Times")


  subcon_fao_wc <- tm_shape(subcon) + #basemap
    tm_fill(col = "#FFFFFF") +
    tm_shape(raster::crop(countries, subcon)) + #countries
    tm_borders(col = "#CCCCCC") +
    tm_shape(raster::crop(fao_wc, subcon)) + #FAO Worldclim
    tm_fill(col = "#33638D") +
    tm_shape(raster::crop(plots, subcon)) + #TDF plots
    tm_symbols(size = 0.025, shape = 21, col = "#CC0000",
               border.lwd = 0.5, border.col = "#CCCCCC") +
    tm_add_legend("symbol", col = "#CC0000",
                  shape = 16, size = 0.75,
                  labels = "Tropical Dry Forest Plots") +
    tm_add_legend("symbol", col = "#33638D",
                  shape = 15, size = 0.75, #sizing helps stack symbols
                  alpha = 0.8, labels = "FAO, Worldclim") +
    tm_layout(bg.color = "#A6CEE3", #ocean
              main.title = " ",
              main.title.position = 0,
              inner.margins = c(0.3, 0, 0, 0),
              legend.position = c(0.01, 0.05), #coords (left, bottom)
              legend.text.size = 0.5, #sizing helps stack text
              legend.width = -1, #prevents any auto re-sizing
              fontfamily = "Times")


  subcon_dry_wc <- tm_shape(subcon) + #basemap
    tm_fill(col = "#FFFFFF") +
    tm_shape(raster::crop(countries, subcon)) + #countries
    tm_borders(col = "#CCCCCC") +
    tm_shape(raster::crop(dry_wc, subcon)) + #Dryflor Worldclim
    tm_fill(col = "#33638D") +
    tm_shape(raster::crop(plots, subcon)) + #TDF plots
    tm_symbols(size = 0.025, shape = 21, col = "#CC0000",
               border.lwd = 0.5, border.col = "#CCCCCC") +
    tm_add_legend("symbol", col = "#CC0000",
                  shape = 16, size = 0.75,
                  labels = "Tropical Dry Forest Plots") +
    tm_add_legend("symbol", col = "#33638D",
                  shape = 15, size = 0.75, #sizing helps stack symbols
                  alpha = 0.8, labels = "Dryflor, Worldclim") +
    tm_layout(bg.color = "#A6CEE3", #ocean
              main.title = " ",
              main.title.position = 0,
              inner.margins = c(0.3, 0, 0, 0),
              legend.position = c(0.01, 0.05), #coords (left, bottom)
              legend.text.size = 0.5, #sizing helps stack text
              legend.width = -1, #prevents any auto re-sizing
              fontfamily = "Times")


  #save maps with insets
  tmap_save(subcon_ml_wc, paste("maps/sub_wc_1.png", sep = ""),
            width = 7/3, height = 2, units = "in", asp = 0)
  tmap_save(subcon_fao_wc, paste("maps/sub_wc_2.png", sep = ""),
            width = 7/3, height = 2, units = "in", asp = 0)
  tmap_save(subcon_dry_wc, paste("maps/sub_wc_3.png", sep = ""),
            width = 7/3, height = 2, units = "in", asp = 0)


  #call maps back in and save
  pnglist = lapply(list.files("maps", pattern = "sub_wc_*", full.names = T), png::readPNG)
  groblist = lapply(pnglist, grid::rasterGrob)
  sub_wc <- gridExtra::grid.arrange(grobs = groblist, nrow = 1)
  ggsave(paste("maps/tdf_fig7_", i, ".png", sep = ""),
         sub_wc, width = 7, height = 2, units = "in")


 }
} #Worldclim
```

References

Banda, K., Delgado-Salinas, A., Dexter, K. G., Linares-Palomino, R., Oliveira-Filho, A., Prado, D., … & Weintritt, J. (2016). Plant diversity patterns in neotropical dry forests and their conservation implications. Science, 353(6306), 1383-1387.

Fick, S. E., & Hijmans, R. J. (2017). Worldclim 2: new 1-km spatial resolution climate surfaces for global land areas. International journal of climatology, 37(12), 4302-4315.

Gorelick, N., Hancher, M., Dixon, M., Ilyushchenko, S., Thau, D., & Moore, R. (2017). Google Earth Engine: Planetary-scale geospatial analysis for everyone. Remote Sensing of Environment, 202, 18-27.

Hansen, M. C., Potapov, P. V., Moore, R., Hancher, M., Turubanova, S. A. A., Tyukavina, A., … &

Kommareddy, A. (2013). High-resolution global maps of 21st-century forest cover change. science, 342(6160), 850-853.

Hoffman, M., Koenig, K., Bunting, G., Costanza, J., & Williams, K. (2016). Biodiversity Hotspots (Version 2016.1) [Data set]. Zenodo. http://doi.org/10.5281/zenodo.3261807.

Huffman, G.J., E.F. Stocker, D.T. Bolvin, E.J. Nelkin, Jackson Tan. Andrey Savtchenko. 2019-03-20. GPM_3IMERGDF. Version 06. GPM IMERG Final Precipitation L3 1 day 0.1 degree x 0.1 degree V06. Greenbelt, MD. Archived by National Aeronautics and Space Administration, U.S. Government, Goddard Earth Sciences Data and Information Services Center (GES DISC). https://doi.org/10.5067/GPM/IMERGDF/DAY/06. https://disc.gsfc.nasa.gov/datacollection/GPM_3IMERGDF_06.html. Digital Science Data.

Holdridge, L. R. (1967). Life zone ecology. Life zone ecology., (rev. ed.)).

Karger, D.N., Conrad, O., Böhner, J., Kawohl, T., Kreft, H., Soria-Auza, R.W., Zimmermann, N.E., Linder, H.P. and Kessler, M., 2017. Climatologies at high resolution for the earth's land surface areas. Scientific data, 4, p.170122.

Murphy, P. G., & Lugo, A. E. (1986). Ecology of tropical dry forest. Annual review of ecology and systematics, 17(1), 67-88.

Natural Earth (2019). 1:10m Cultural Vectors, Admin 0 - Countries. Available at: https://www.naturalearthdata.com/about/terms-of-use/ (accessed on 12 April 2020).

Olson, D. M., Dinerstein, E., Wikramanayake, E. D., Burgess, N. D., Powell, G. V., Underwood, E. C., ... & Loucks, C. J. (2001). Terrestrial Ecoregions of the World: A New Map of Life on EarthA new global map of terrestrial ecoregions provides an innovative tool for conserving biodiversity. BioScience, 51(11), 933-938.

Sunderland, T., Apgaua, D., Baldauf, C., Blackie, R., Colfer, C., Cunningham, A. B., ... & Ickowitz, A. (2015). Global dry forests: a prologue. International Forestry Review, 17(2), 1-9.

Title, P.O., Bemmels, J.B. (2018). ENVIREM: an expanded set of bioclimatic and topographic variables increases flexibility and improves performance of ecological niche modeling. Ecography. 41:291–307.

Trabucco, A., & Zomer, R. J. (2018). Global aridity index and Potential Evapo-Transpiration (ET0) Climate Database v2. CGIAR Consortium for Spatial Information (CGIAR-CSI). Available online: https://figshare.com/articles/Global_Aridity_Index_and_Potential_Evapotranspiration_ET0_Climate_Database_v2/7504448/1 (accessed on 25 June 2019).
