# Supplementary material for: Global tropical dry forest extent and cover: A comparative study of bioclimatic definitions using two climatic data sets
Source: PLoS One. 2021 May 20;16(5):e0252063. doi: 10.1371/journal.pone.0252063 (PMC8136719; doi:10.1371/journal.pone.0252063)
Supplement: Attachment — Submitted filename: Reviewer-1 comments.pdf [file pone.0252063.s007.pdf]

Review of the manuscript PONE-D-20-37820

The manuscript is an interesting address of the potential extent of the tropical dry forest biome based on bioclimatic definitions and climatic data sets to improve global estimates of distribution, cover, and change over time.

However, I have certain concern about:
-The authors try to do many comparisons in this manuscript but statistically there was no test used to do the real comparisons.
-Validation issue is important to be highlighted and there is lack of validation especially the ground validation.
-The authors need to explain with more details, why they used WorlClim and CHELSA Climatologies.
-More information about vegetation and plant cover is needed within study area.
-Variability in selected plots with different sites and validation is problematic, the authors failed to consider and state the variability between the study sites, plots and locations, so the authors need to show that statistically (i.e, residual plots).
-For the comparison within WWF Ecoregions and field plots, the authors need to show the accuracy level of the field plot location and within what range?
-Table 5. is showing the best methods for estimating closed canopy (>40% close), what about open canopy?
-There is lack of information about tree species and density.
- Abbreviations in the text are confusing and need to be simple.
- The objectives are not clear and need to be more specific with some hypothesis and expectation for each objective.
- Discussion part needs to be more specific related to the results.

Other Comments:
Title: Add “using two climatic data set” :

Global tropical dry forest cover and extent: A comparative study of bioclimatic definitions using two climatic data sets.

Introduction:
Line 63-65: need more details
Line 69-71: need citation.
Materials and Methods:
- Data collection needs more clarification
